# Critical diversity: Divided or united states of social coordination

**DOI:** 10.1371/journal.pone.0193843

**Published:** 2018-04-04

**Authors:** Mengsen Zhang, J. A. Scott Kelso, Emmanuelle Tognoli

**Affiliations:** 1 Center for Complex Systems and Brain Sciences, Florida Atlantic University, Boca Raton, Florida, United States of America; 2 Intelligent System Research Centre, Ulster University, Derry ~ Londonderry, Northern Ireland; Consejo Nacional de Investigaciones Cientificas y Tecnicas, ARGENTINA

## Abstract

Much of our knowledge of coordination comes from studies of simple, dyadic systems or systems containing large numbers of components. The huge gap ‘in between’ is seldom addressed, empirically or theoretically. We introduce a new paradigm to study the coordination dynamics of such intermediate-sized ensembles with the goal of identifying key mechanisms of interaction. Rhythmic coordination was studied in ensembles of eight people, with differences in movement frequency (‘diversity’) manipulated within the ensemble. Quantitative change in diversity led to qualitative changes in coordination, a critical value separating régimes of integration and segregation between groups. Metastable and multifrequency coordination between participants enabled communication across segregated groups within the ensemble, without destroying overall order. These novel findings reveal key factors underlying coordination in ensemble sizes previously considered too complicated or 'messy' for systematic study and supply future theoretical/computational models with new empirical checkpoints.

## Introduction

The function of living systems (e.g. brain, human society, ecosystem) depends on the coordination of multiple components and processes. Such coordination depends on intrinsic characteristics of the interacting entities as well as the form of interaction between them [1–4]. Living systems exhibit a myriad of rhythmic behaviors [5], e.g. humans with their daily, weekly, monthly routines [6] and physiological rhythms [7]; brains with their waves [8]; and species with their life-cycles [9]. By virtue of its temporal symmetry (i.e. translational symmetry in time), rhythmic coordination serves as a fine soil for experimental and theoretical study of laws of interaction between components of dynamical systems. The study of two interacting entities has laid experimental and theoretical foundations for addressing how coordinative structures form, adapt and change. Whether it is humans coordinating with sensory stimuli [10,11], coordinated movements within the same person [12–15], between two persons [16–22], two neuronal populations [23,24], humans and machines [25–27], or humans and other species [28,29], similar tendencies to form or learn certain relative phase and frequency patterns have been observed. Essential phase patterns, their stabilities and transitions have been well described mathematically in terms of informationally coupled dynamical systems [30–33]. A little beyond dyads, triadic and tetradic coordination have been studied mainly in animal gaits or multilimb movements with a richer repertoire of patterns–combinations of dyadic patterns satisfying certain symmetry constraints [34–38]. Beyond systems with a relatively small number of interacting components, the focus of interest leaps toward systems of much larger scales–e.g. flashing fireflies [39], neuronal populations [40], or the clapping of an ardent audience [41]–whose sheer size eludes detailed investigational techniques but favors low-dimensional measures at coarser scales (e.g. collective synchronization). Such synchronization has been reproduced in various coupled oscillator models, e.g. [42–45].

Despite this gap between systems of very few and very many components (with rare exceptions, [46]), daily social interaction often unfolds in the middle, for example, coordinating with a group of colleagues at work, or afterwards engaging in a variety of gatherings with friends and families, or various forms of folk dancing and Ceilidhs. The choice of the number of independently manipulatable components goes hand in hand with available paradigms for approaching coordination phenomena. With very few components, the repertoire of collective patterns and phase transitions can be fully explored with the help of experimental manipulation and theoretical models, but the limited size may curtail the complexity of spatial organizations. With very many components, possible coordination patterns (described at a microlevel) become too numerous to be studied exhaustively (due to high dimensionality of the phase space); the large number of components also makes it difficult to utilize systematic manipulations to carry the system through its repertoire of possible patterns. Instead, low-dimensional (macro) measures such as the overall level of synchronization can serve as an order parameter to capture collective states of the system [1,43]. As important as such descriptions of coordination are, macro measures meet their limit when one attempts to characterize the system’s organizational complexity. Under the broad umbrella of “incoherent” states, what are the possible organizations? How can we explore such organizations systematically in the laboratory? To answer these kinds of questions, a way is needed to experimentally manipulate the system’s coordination dynamics on multiple spatial and temporal scales of description. We chose an ensemble of intermediate size (N = 8 people) operating under the assumption that this is big enough to reveal the system’s organizational complexity, yet small enough to yield to experimental manipulation. Our strategy was to bridge this two-fold gap of system size and experimental control.

We studied rhythmic movement coordination in ensembles of eight people who were predisposed to move at the same or different frequencies. Existing empirical findings and theories suggest that the form and stability of coordination varies with the strength of coupling and the difference in natural frequency (frequency predisposition) between components [11,32,47]. On this basis, we hypothesized that manipulating the distribution of frequency predispositions and coupling strength should produce different propensities for coordination, and induce different forms of collective behavior. Because it is possible to control systematically and measure quantitatively, frequency difference was chosen as a parameter to manipulate diversity within and between group members. We wanted to know how different diversity conditions favor the formation, persistence and change of multiple groups that are potentially integrated within themselves but segregated between each other.

## Results

Fifteen independent ensembles of eight people (N = 120) participated in the study (for details see Materials and Methods). All were instructed to tap rhythmically on a touchpad. At the beginning of each trial, members of an ensemble were each paced with a metronome; after the pacing period, they were able to see each other’s taps as flashes (dubbed “human fireflies”) on an array of LEDs situated at eye height in front of them. The task was to keep tapping at one’s own metronome frequency (tempo) throughout the entire trial. No instructions were given to coordinate with others.

To study how patterns of coordination among participants may form or dissolve, we introduced different levels of diversity by manipulating the assignment of metronomes to each participant. The metronomes divided the participants into two groups of four with frequency difference (*δ f*, also referred to as level of diversity below) of either 0 Hz (1.5 vs. 1.5 Hz), 0.3 Hz (1.35 vs. 1.65 Hz), or 0.6 Hz (1.2 vs. 1.8 Hz). Within each group the four participants were paced at the same frequency. Overall, participants followed the metronome frequency during both pacing and interaction phases, in accord with instructions (see Section E in S1 File). In the following sections, we demonstrate the main findings, which may be best read along with the extended quantitative and theoretical analyses provided in the Supporting Information (S1 File).

### Spontaneous phase coordination and spatiotemporal metastability

**Fig 1:**
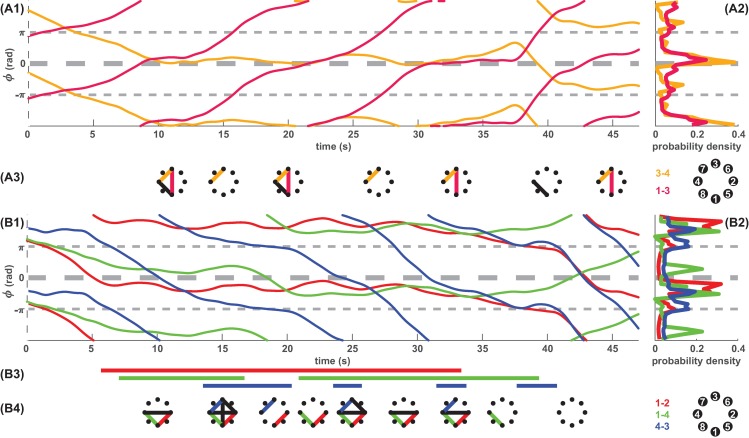
Coordination dynamics of phase relations among multiple agents. (A1) Exemplary relative phase trajectories show the metastable phase coordination of three persons (2 moduli plotted to help perceiving trajectories’ temporal continuity). Shortly into the interaction stage (10s), dyad 3–4 coordinated near inphase for 25s (relative phase *ϕ ≈ 0* orange, flattening of *ϕ* trajectories indicates phase coordination, or dwells), then switched into a pattern near antiphase (*ϕ ≈ ±π* orange, 40-47s). Dyad 1–3 also dwelled around inphase but for shorter durations (A1, red curve flattening around 12, 22, 32s). The interaction shows tendencies for bistability (inphase and antiphase), as also seen in the histograms of the relative phase (A2), with the orange distribution more pronounced at antiphase than the red. (A3) shows the spatial organizations of phase coordination among agents 1, 3, and 4 at moments corresponding to the time-axis in (A1; for interpretation see B4 below). (B1-4) shows an example of four-person interaction in similar format to the above. Dynamics of *ϕ* (B1) reveals phase coordination on various time scales, visualized in (B3) where the length of a bar annotates the duration of phase dwell between a pair of participants. Dyad 1–2 (red) showed the longest dwell, Dyad 1–4 (green) a bit shorter, and Dyad 4–3 (blue) the shortest. The coexistence of multiple timescales of coordination gives rise to a constantly evolving spatial organization of the group, shown as a sequence of graphs in (B4) where each node presents a participant and an edge indicates phase dwell (color coding corresponds to B1-3, black edges are dyadic dwells whose dynamics are not shown in B1-2; coordination within the other group, i.e. agents 5, 6, 7, 8, is not shown for reasons of clarity).

The dynamics of relative phase between participants revealed that the participants spontaneously coordinated in various phase patterns and switched between them, despite not being given any instruction to do so. Such dynamic patterns are exemplified in Fig 1A1–1A3 which shows a trial of interaction among three persons (labeled with numbers 1, 3 and 4, reflecting spatial location on LED arrays, see legends under A2). The evolution of their relations is shown in (A1) as trajectories of dyadic relative phase (*ϕ*, reported in radians throughout this paper) for pairs 3–4 (orange) and 1–3 (red). When a trajectory is horizontal, the pair is strongly coordinated by holding an (almost) constant phase relation (termed phase locking or dwell); when the trajectory is tilted, the pair is uncoordinated (phase wrapping). Dyad 3–4 (orange) engaged in a long dwell at inphase (*ϕ ≈* 0, 10-35s in A1, largest peak in A2), then switched to a near antiphase pattern (*ϕ≈ π*, 40s onward in A1, small peak in A2). Such near inphase/antiphase patterns are signs of bistability widely observed in biological coordination [48]. Dyad 1–3 (red) also coordinated near inphase but in much briefer and recurrent dwells (around 10, 20, 30s in A1, largest red peak in A2), interleaved with escapes from it. This type of intermittent or relative coordination [49] characterized by consecutive epochs of dwells and escapes corresponds to the metastable regime in models of coordination dynamics [3,50]. Evidence for metastabilty was often seen in single trial dynamics (see Section J in S1 File for a statistical approach). Besides bistable and metastable coordination observed within specific pairs of participants, a higher order interaction becomes apparent when we examine the two pairs together: during the long dwell of Dyad 3–4, three epochs of phase shift (bumps in orange curve at 15, 25, 35s in A1) followed precisely after each dwell of Dyad 1–3 (red). Moreover, as each dwell of Dyad 1–3 became longer than the previous one, the phase shift in Dyad 3–4 became bigger, to the point where the shift was so big (38s) that Dyad 3–4 broke up their predominant inphase pattern and switched to antiphase. This finding indicates that the joining of a new member (e.g. person 1) induced changes in preexisting coordinative relations (e.g. Dyad 3–4), strongly suggesting that multiagent coordination is more than the sum of isolated dyads (see Section H in S1 File for a statistical analysis). As an aid to visualization, the spatial arrangement corresponding to the foregoing temporal changes are illustrated in A3.

In the experiment, epochs of phase coordination were mostly transient or intermittent (i.e. metastable dwells), covering a wide range of time scales, with a mean duration of 4.64s (± 4.04s) and a long tail of more persistent phase patterns up to the entire duration of interaction, about 50s (See Fig B in S1 File for distribution). The confluence of metastability and multiple coupled agents allows the coexistence of multiple time scales of coordination in a group, as Fig 1A1–1A3 already hinted (orange–long dwell, red–short dwells with more frequent recurrence). Multiple coordinative time scales allow different members of a group to come together at different times, thus allowing the group to visit a variety of spatial patterns at different times. An example of four-person interaction is given in Fig 1B1–1B4 illustrated as three dyadic relative phases (dynamics in B1, distributions in B2). The duration of phase dwells is marked in (B3): red dyad with a long dwell, green dyad a bit shorter, and blue dyad even shorter. Such multiplicity in the time scale of metastable coordination led the four-person group through a variety of spatial patterns from moment to moment (B4) rather than to persist as a static structure (which would be the case if, e.g., phase coordination were absolutely stable). Thus, in the present case of intermediate sized group arrangements, spatiotemporal metastability—coexisting tendencies for integration and segregation—is rather more characteristic of coordination than collective synchronization [50,51].

### Dominant patterns of coordination and their relation to diversity

**Fig 2:**
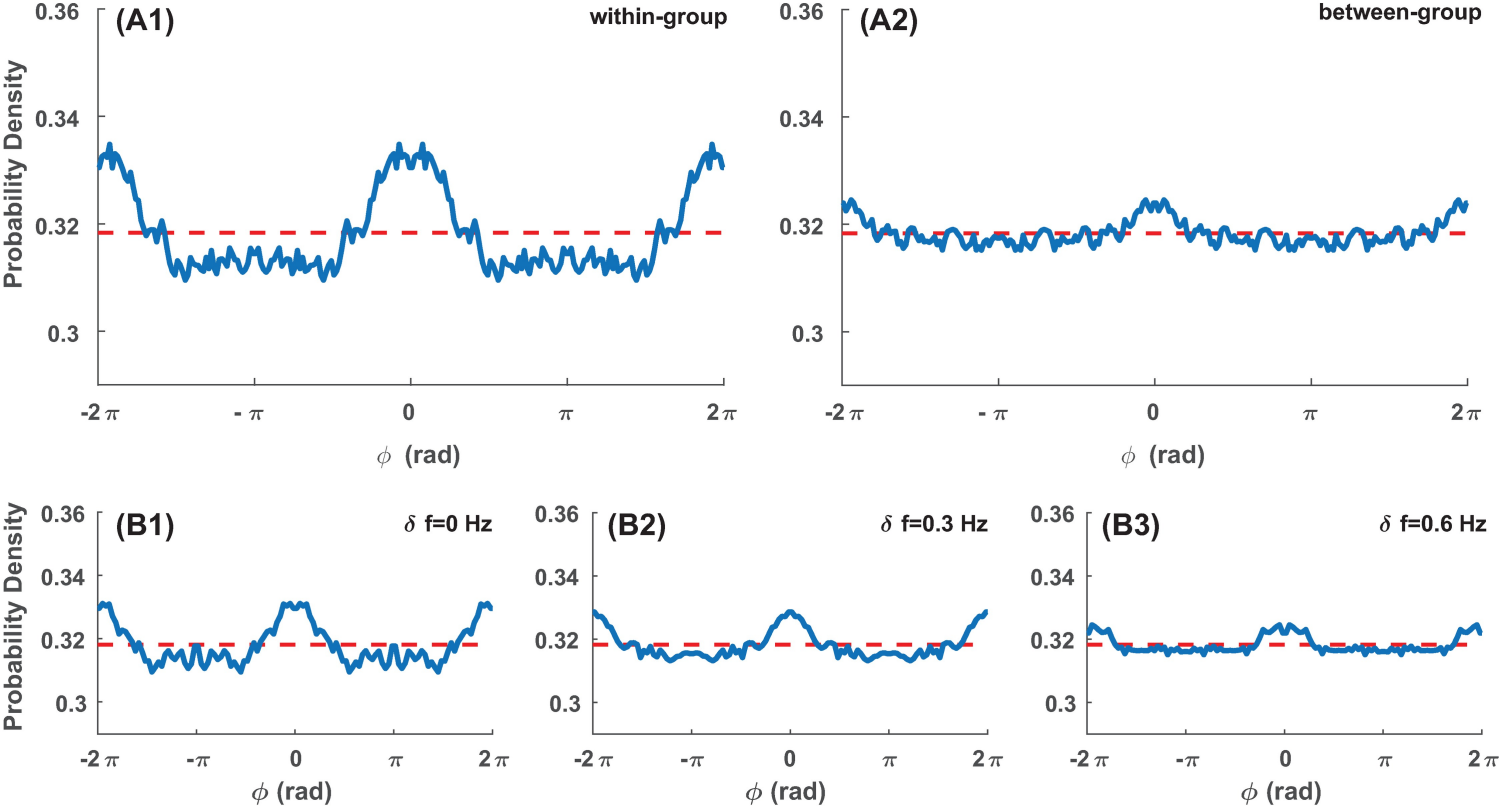
Aggregate distributions of phase relations. Blue solid lines are distributions of relative phases in the experiment (histograms were computed and statistically tested in the interval [*0*, *π*] then repeated in the interval [*-2π*, *2π*] for visualization). Red dashed lines correspond to chance level (uniform) distribution. (A1) shows relative phase between members within the same frequency group, (A2) between different groups, (B1-3) for ensembles with diversity level *δ f* = 0, 0.3, 0.6Hz respectively. Inphase (central peak) is clearly a dominant pattern throughout, but its dominance diminishes with the diversity parameter displayed in (B1-3). Inphase preference was more pronounced within-group (A1), where participants shared the same initial frequency, than between-group, where frequency diversity was introduced (A2).

When all phase relations were considered in aggregate, we found that inphase coordination was clearly a dominant phase pattern (central peaks in distributions of relative phase *ϕ* in Fig 2). Yet this dominance of inphase depended on both local and global diversity. Inphase was more dominant locally within a group (participants paced at the same frequency) than between groups (where diversity was introduced by *δ f*; Fig 2, A1, probability density for within-group *ϕ* significantly above chance from 0 to 0.24π, A2, for between-group *ϕ* significantly above chance from 0.05π to 0.08π, at $\hat{p}$ <0.05, where ‘hat’ denotes Bonferroni correction for multiple comparison throughout the text; see Section G in S1 File for confidence intervals for chance level distribution). Globally, the dominance of inphase in the entire ensemble decreased as diversity increased (B1-3 for *δ f* = 0, 0.3, 0.6 Hz respectively: B1 significantly above chance from 0 to 0.14π, B2 from 0 to 0.09π, $\hat{p}$ <0.05; B3 n.s.). This suggests that inphase coordination is an important characteristic for the formation and maintenance of coordinative structures regardless of group size, especially when diversity is low. Considering only epochs of strong coordination (dwells), we found a wide range of phase relations, where antiphase, along with inphase, was also a preferable phase relation (for details see Section F in S1 File).

**Fig 3:**
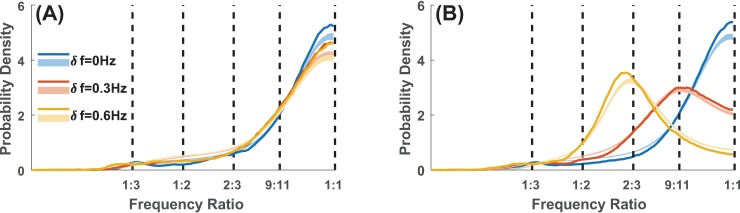
Multifrequency coordination. Ensembles with low diversity were dominated by 1:1 coordination, while ensembles with high diversity also steered towards higher-order ratios. Solid lines show the probability density of frequency relations within- (A) and between-group (B) for the 3 diversity conditions (color coded). Thin shaded areas (with corresponding colors) are confidence intervals for null distributions (p<0.0005 for each of 100 bins, corresponding to $\hat{p}$ <0.05 for an entire distribution using Bonferroni Correction; generated from randomly permutated taps, which represent the expected distribution from non-interacting agents tapping at required frequencies). For within-group relations (A), the peaks at 1:1 are far above chance, indicative of stabilizing phase relations at the same frequency. For between-group relations (B), low to moderate diversity (blue, red, *δ f* = 0, 0.3 Hz) led to above-chance coordination at 1:1; in contrast, for high diversity (yellow, *δ f* = 0.6, corresponding to metronome ratio 2:3), coordination was below chance at 1:1 but far above chance at a higher order ratio near 2:3.

Beyond patterns of phase relations, other types of coordination were observed. One of them is a form of multifrequency coordination that binds behavior at different frequency ratios [14,15,52,53]. We studied which frequency ratios constitute preferred coordination patterns by comparing their probability density to chance levels (computed from randomly permuted taps, see Section D in S1 File for details). Chance level distributions reflect expected occurrence of different frequency ratios as a result of participants’ maintaining metronome frequencies without interacting with each other. Hence, we expected chance level distributions to peak around ratios corresponding to the three diversity conditions, i.e. 1:1 (*δ f* = 0 Hz), 9:11 (*δ f* = 0.3 Hz), and 2:3 (*δ f* = 0.6 Hz). Fig 3 shows the distribution of instantaneous frequency ratios in terms of within-group (Fig 3A) vs. between-group (Fig 3B) coordination for different levels of diversity (blue *δ f* = 0 Hz, red *δ f* = 0.3 Hz, yellow *δ f* = 0.6 Hz). A frequency ratio is a preferred coordination pattern if its probability density (solid lines) is above chance level (light-color bands). Within-group participants coordinated primarily at 1:1 ratio (Fig 3A, all $\hat{p}’s$ <0.05), which is consistent with the high level of phase-locking reported above. For between-group coordination (Fig 3B),1:1 was still the preferred ratio when there was no diversity (*δ f* = 0 Hz, $\hat{p}$ <0.05); a higher order ratio near 2:3 was preferred when the diversity was large (*δ f* = 0.6 Hz; $\hat{p}$ <0.05). For intermediate diversity (*δ f* = 0.3 Hz), the between-group frequency coordination was barely above chance at metronome ratio 9:11 (for metronomes at 1.35 Hz and 1.65 Hz), but significantly more concentrated than chance near 1:1 ($\hat{p}$ <0.05). In short, under appropriate diversity conditions, lower order (1:1) and higher order (e.g. ~2:3) frequency coordination can coexist–a basis for complex spatiotemporal coordination. Furthermore, this type of coordination with frequency ratios (one which is less straightforward to detect and less studied) is specific to between-group interactions.

### Segregation and integration of groups: Critical diversity

**Fig 4:**
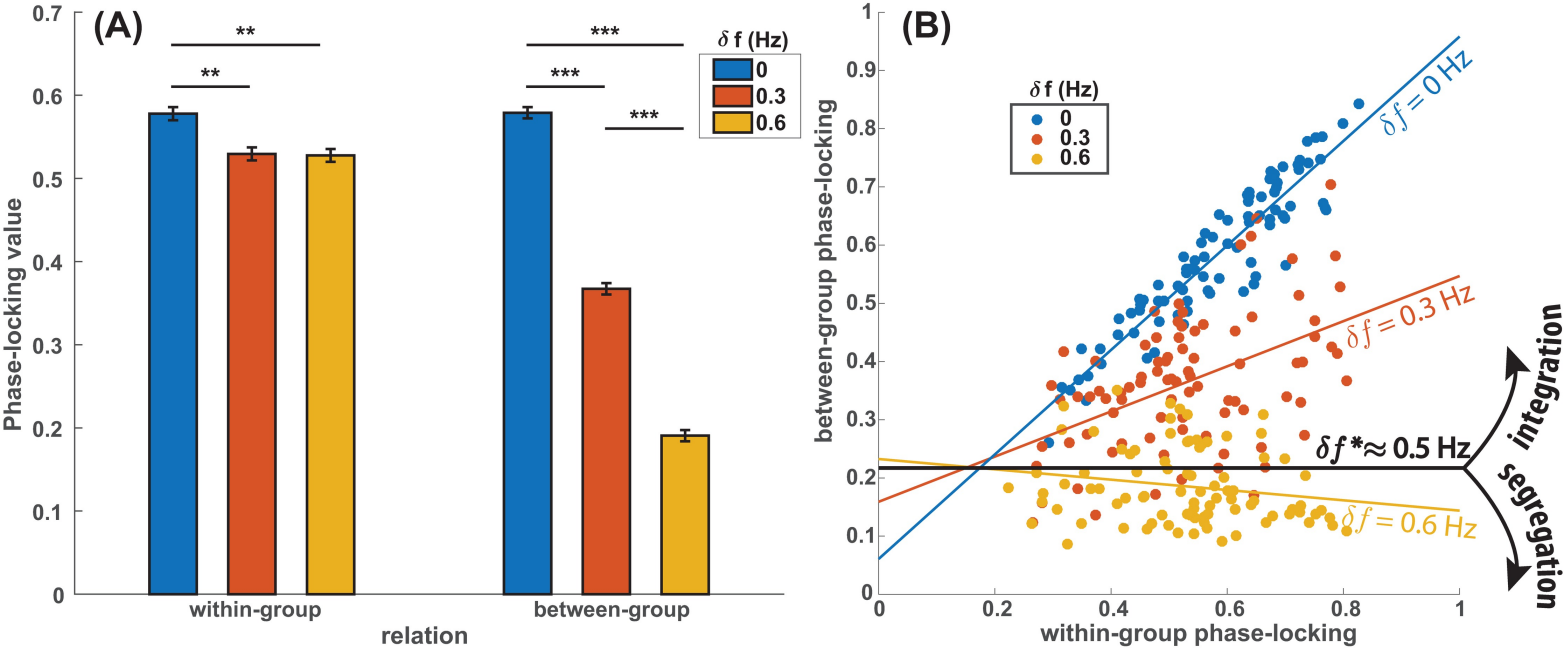
Diversity parametrically controls integration~segregation of groups within ensembles–the emergence of spatial scales. (A) Phase locking between groups decreased monotonically when between-group *δ f* increased (A, right). Within groups however (A, left), where agents’ initial frequencies were uniform, phase locking was still affected by the presence of another group of a different frequency (red, yellow bars significantly lower than blue bar), demonstrating that interactions are sensitive to the multiagent context in which they are embedded. (** p<0.01; *** p<0.001; error bars represent standard errors) (B) A scatterplot reveals linear associations between phase locking within- and between-group (each point represents a trial), whose slopes were modulated by the diversity parameter *δf* (denoted by color, see legend). Linear regressions had positive slope for lower diversity (blue and red colored lines) indicating integration of initial groups into larger coordinative structures, while a negative slope was found for the largest diversity (yellow line), indicating intergroup segregation. A critical parameter of diversity (δ f*) was identified that borders the regimes of integration and segregation (black line).

Having studied coordination at the micro level (person to person), we turn now to the macro level of integration and segregation between groups. In order to do so, we first quantified coordination as the level of phase locking between individuals from the same and different initial groups (i.e. within- and between-group coordination respectively). Fig 4A shows the average results. We found that as initial frequency difference between groups (*δ f*) increased, phase-locking between groups weakened dramatically (Fig 4A, right cluster). Interestingly, phase-locking within groups (no diversity within-group by design) was also weakened by virtue of the difference with the other group (Fig 4A, left cluster, notice orange and yellow bars significantly shorter than blue; MANOVA, interaction effect, F(2,7246) = 198.2, p<0.001; see Section L in S1 File for MANOVA main effects analysis). That is, local coordination (e.g. within group) was influenced by the larger context (difference with other groups), as exemplified also in Fig 1A.

Next, we quantified group-level segregation~integration by studying the relation between within-group and between-group coordination. If more within-group coordination leads to more between-group coordination, the groups may be said to become integrated. If more within-group coordination leads to less between-group coordination, the groups may be said to become segregated. In Fig 4B, for the zero intergroup difference (*δ f* = 0 Hz, blue dots), a large value of within-group phase-locking is paired with a large value of between-group phase-locking, indicating that the initial groups have merged. The same is true, though to a lesser extent, for *δ f* = 0.3 Hz. For *δ f* = 0.6 Hz, however, a larger value of within-group phase-locking is associated with a smaller value of between-group phase-locking, suggesting that stronger coordination within the group prevents coordination with members of the other group, or conversely, switching to another group reduces the coordination with one’s original group. Quantitatively, for small diversity (*δ f* = 0, 0.3 Hz), initial groups integrated into one supergroup, as seen from the positive slope of regression lines (Fig 4B, blue, red; *β*_*1*_^*0 Hz*^ = 0.88, t(84) = 20.0, p<0.001; *β*_*1*_^*0*.*3 Hz*^ = 0.31, t(84) = 3.94, p<0.005). For larger diversity (*δ f* = 0.6 Hz), the groups became more segregated (negative slope; Fig 4B, yellow; *β*_*1*_^*0*.*6 Hz*^ = -0.14, t(85) = -2.83, p<0.01).

To estimate the critical diversity that marks the boundary between integration and segregation, we regressed the degree of integration *β*_*1*_^*δ f*^ against the intergroup difference *δ f*. We found a significant negative linear relation between those variables (linear regression, *α*_*0*_ = 0.86, t(1) = 20.5, p<0.05; *α*_*1*_ = -1.70, t(1) = -15.7, p<0.05). By finding when integration vanishes (*β*_*1*_^*δ f*^ = 0), we identified a critical frequency difference (*δ f* ^*^) of 0.5 Hz as a boundary between the two different macro-organizations, i.e. a critical value that distinguishes segregation and integration.

### Segregation and transitions of spatial order

**Fig 5:**
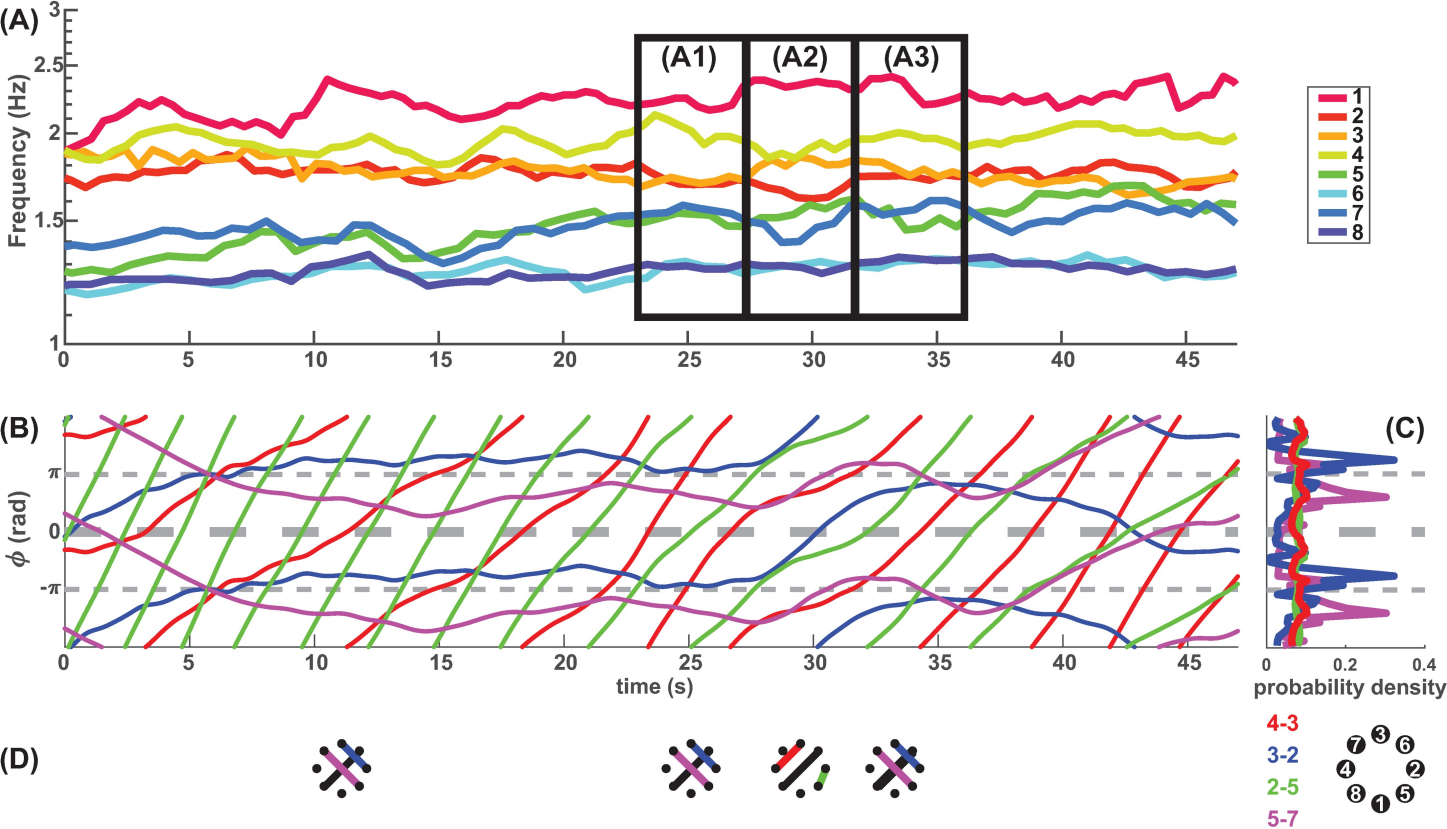
Frequency diversity contributes to spatial organization and reorganization. (A) Instantaneous frequencies of an ensemble of eight interacting agents (smoothed by averaging four consecutive taps). Agents 1 to 4 (warm colors) were paced with the same metronome frequency 1.8 Hz, and similarly agents 5 to 8 (cold colors) were paced at 1.2 Hz, (i.e. *δ f* = 0.6 Hz), which helped create two initial frequency groups. Soon after the beginning of the interaction (~12s, corresponding to the first graph in D), initial groups divided into five local structures: three pairs (3–2, 5–7, 6–8) and two individuals (agent 1 largely independent, agent 4 oscillating between agent 1 and pair 3–2). The frequency pairing held up to the time of (A1), then a sudden reorganization occurred from (A1) to (A2)–an exchange of partners (3–2 broke up and recoupled into 4–3, 2–5; 7 left alone; corresponding to the 3^rd^ graph in D). The new pairing lasted a few seconds then returned to a similar organization to (A1) at the time of (A3). Phase relations of the pairs involved in the reorganization (A1-3) are illustrated in (B) as time series and in (C) as distributions of four dyadic relative phases. The new organization at A2 lasted exactly the time for pair 3–2 (blue) to break up an antiphase relation (27s) then return to it (33s) after phase wrapping for one cycle. This transition in phase relations corresponds closely to the transitions of frequency grouping. To visualize the spatial consequences of such phase/frequency regrouping, graphs in (D) were used as representations of the coordinative structure. Each node represents a participant at the actual location of the LED representing that participant (up to rotation). Each edge represents the existence of strong phase coordination between two participants at the time (aligned with x-axis in B). The spatial reorganization is apparent from the 2^nd^ and 3^rd^ graph aligned to (A1) and (A2) respectively. Interestingly, the 3^rd^ graph, albeit distinct from the rest, is in fact isomorphic to the other graphs.

We now return to real time dynamics to unpack the meaning of macro-level “segregation” in the foregoing statistical conclusion. In an example shown in Fig 5, the ensemble was initially divided into two frequency groups (early on in Fig 5A; faster group of agents 1 to 4, slower group of agents 5 to 8), thanks to the large difference between their metronome frequency (*δ f* = 0.6 Hz). Soon the ensemble developed into multiple local structures which were coordinated within and segregated between each other (three pairs 3–2, 5–7, 6–8, and two individuals 1, 4; this spatial order can be easily seen in D, first two graphs, 10-25s). The large initial diversity allowed the coexistence of multiple segregated groups and enabled the ensemble to form a sustained spatial order by providing sufficient frequency isolation between local structures (in contrast to the low diversity scenario where spatial patterns go through constant reorganization, e.g. Fig 1, Fig H in S1 File). However, a segregated spatial order does not have to be static. To the contrary, there was a sudden transition from one segregated spatial order (A1 and 2^nd^ graph in D) to another, also segregated, spatial order (A2 and 3^rd^ graph in D, a period marked with multiple partner exchanges), then back to the original (A3, and 4^th^ graph in D). This kind of micro-level exchange of members across frequency groups has been observed in 77% of the trials in the segregated condition (*δ f* = 0.6 Hz). It suggests that segregation is a macro property of ensembles, sustainable despite the coexistence of dynamical exchanges at micro level.

## Discussion

### Integration and segregation in a diverse group

Rhythmic coordination is ubiquitous in natural systems from the cells of the heart to the neurons of the brain, from fireflies to people [3,39,41,46,54–58]. The convergence of multiple interacting elements to global synchronization has been the focus of experimental and theoretical studies [41–45,59,60]. Behavioral synchronization is known to facilitate social communication and the development of social affection or bonding [61–65], and is important to understanding social coordination dynamics [66,67]. Nevertheless, within a community, people coordinate in multiple social groups at various spatiotemporal scales–a complex organization that is far from uniform synchronization [68–70]. In fact, the components of living systems often compartmentalize into distinct communities or modules, highlighted by dense interactions within communities and loose interactions between communities [71,72]. This form of organization, embracing both integration and segregation among its elements, can lead to greater persistence and robustness of the system [73–76], and influence structural and functional complexity depending on the scale of integration [77–79]. Investigation of the conditions leading to the formation, change, and dissolution of segregated structures is a necessary step to understanding and controlling complex systems.

We demonstrated experimentally how coexisting groups integrated and segregated in an ensemble of eight interacting people. Each half of the ensemble was predisposed to move at a distinct frequency prior to social interaction, thereby creating two initial frequency groups with a controllable parameter of diversity between them (*δ f*). People engaged in more phase coordination with those who were predisposed to move at the same frequency than with those who performed at a different frequency (Fig 4A; Fig K in S1 File left). This is a form of “homophily”–people prefer interacting with those who are similar to themselves than with those who are different [80]–known to contribute to segregation in diverse communities [81–84]. Indeed, the integrating force of sameness is complemented by the segregating force of difference [85].

To what extent do quantitative changes in intergroup diversity induce a qualitative change in intergroup relationships? We have shown that low-to-moderate diversity led to integration of the groups (*δ f* = 0, 0.3 Hz; Fig 1B): more coordination within-group was associated with more coordination between-group. High intergroup diversity led to segregation (*δ f* = 0.6 Hz; Fig 1B): more coordination within-group was associated with less coordination between-group. Parametrically varying diversity made it possible to estimate the critical value of diversity (*δ f* ^*^): exceeding this critical value led to segregation; remaining below the critical value led to integration. Identifying the critical values of a dynamical system empirically proves to be a valuable step in many situations, not only to provide essential information on the organizing principles and potential behaviors of the system, but also to serve as key phenomena to be reproduced in theoretical models [86,87].

A complex system consists of interactions at multiple spatial scales, where activities at one scale are connected with those of another scale [88,89]. How the macro environment constrains micro activities was illuminated by comparing dyadic interactions embedded in a group with expected behavior of dyads in isolation. If dyads (micro) were not influenced by the larger environmental context (macro), the same amount of coordination would be observed within groups at all three levels of intergroup diversity. The data say otherwise: phase locking within a group was in fact weakened by intergroup diversity (Fig 4A, left). This shows that when a system has multiple components, dyadic interactions may not be fully understood without taking into account the larger environment or context they are embedded in [4,90,91].

### The patterns of coordination

To further understand the micro dynamics of social interaction, we identified the specific phase patterns people adopted. Overall, we found that inphase was visited significantly more often than other phase relation, yet its prominence diminished with increasing diversity (Fig 2). That is, diversity induced a dispersion of phase patterns. Absolute synchronization between components’ behavior is not always desirable: excessive synchrony may induce pathological collective dynamics [92] or impede complex functions [79,93]. Diversity may come to the rescue. Besides inphase, a preference of antiphase over various other phase relations also stood out in episodes of strong interactions (Fig C in S1 File). The present results resonate with existing studies of human rhythmic coordination [3,30]. When coupling was sufficiently strong, the tendency for two oscillatory components to coordinate inphase or antiphase was found across scales, particularly when the components have similar frequency predispositions [94]. When coupling was sufficiently weak, however, the antiphase pattern was more vulnerable to natural frequency differences [32,47]. Both diversity in frequency predispositions [11] and multiagent environment [35,37,95–97] help engender a variety of phase relations that are neither inphase nor antiphase. The agreement between the statistical properties of the interactive behaviors in an ensemble of eight persons and the dynamic properties of dyadic coordination suggests that dyads remain the most stable unit of spontaneous coordination. Yet how can group coordination be achieved with primarily dyadic interactions? This led us to explore the dynamics of phase relations.

Phase relations do not have to be static, as social coordination often evolves on multiple time scales [68,69,98]. Over the course of interaction, we found that most phase relations only lasted a short period of time (4-5s, Fig B in S1 File). Two partners dwell in a phase relation for a few seconds before a “breakup” or “escape” from that relation, and then re-engage the next time they come across a favorable phase relation (e.g. Fig 1). The recurrent relation embodied by a series of dwells and escapes is characteristic of metastable coordination dynamics [50,94,99]. Theoretically and empirically, metastability occurs in weakly coupled dynamical systems when there is sufficient difference in the components’ frequency predispositions. The combination of symmetry breaking and weak coupling eliminates perfectly stable phase relations which are replaced by intermittent or recurrent phasing. In the present study, quantitative analysis confirms that metastability prevails in all conditions of interaction (Fig J in S1 File). Notice that the sequence of dwells and escapes of phase relations also manifests as oscillations in movement frequency (e.g. Fig G in S1 File). In contrast with stable coordination in which components eventually converge to the same frequency, metastability allows components to visit a range of frequencies while still maintaining “social bonds” via intermittent dwells. When multiple metastable relations coexist in the same group, it becomes possible for a person’s transient escape from an existing relation to be at the same time a dwell in a new relation. This chimeric feature (c.f. [51]) allows members of a community to participate in multiple segregated substructures (e.g. a reading club, and a hiking team) while maintaining both the separability of those substructures and communication between them. Such continuous change of membership helps large communities to persist [100] and increase global level of cooperation [101]. Spatiotemporal metastability in multiple-component systems suits both the intuition of daily social interaction, as well as the dynamic patterns observed in large scale social networks [68].

Phase-locking constitutes a rather strong form of coordination. Such coordination comes at a cost in both time and energy if the partners possess different frequency predispositions: the chasm of frequency difference, jointly or unilaterally, must somehow be crossed. In the present experiment, not all forms of coordination required such costly crossovers. As diversity increased, people from different groups were found to adopt particular frequency relations (or ratios) of higher order (e.g. near 2:3, Fig 3B, yellow) as opposed to converging to a single frequency (1:1). Frequency relations appear in the more familiar context of music as polyrhythms. Theoretical and experimental studies have shown the viability of different frequency ratios: higher order ratios (e.g. 2:5, 3:5) are more difficult to maintain (less stable) than lower order ratios (e.g. 1:3, 2:3) in accordance with so-called Arnold tongue and Farey tree principles [15,53,102,103]. Such frequency relations enable segregated groups to maintain communication between each other, without sacrificing within-group cohesion, thus allowing complex coordinative structures to form. Such cross-frequency communication may serve to integrate local activities over long distance and time scales in complex systems, including the brain [89,104,105].

## Conclusions

Our goal was to elucidate the coordination dynamics of ensembles of eight people, where the ensemble is small enough for systematic manipulation in the laboratory, but not too small as to prevent the unfolding of complex social dynamics (i.e., simple, but no simpler). At the macro level, we studied the integration and segregation of groups and how it affects, at the micro level, dyadic interactions embedded within. A novel finding was that the domains of integration and segregation between groups are demarcated by a critical level of intergroup diversity. Diversity across groups also affected the strength and forms of dyadic coordination within groups. In particular, a metastable form of phase coordination was revealed in which phase relations were intermittent rather than stable, thereby allowing people to switch flexibly between partners as a means of maintaining both diversity and unity. When groups were segregated and phase coordination became difficult, social coordination also took the form of cross-frequency coupling. The present work provides a multiscale portrait of the coordination dynamics among multiple agents, and thereby offers quantitative details and reality checks for modelling social dynamics. The analytical methods used here can be extended to study segregation and integration in larger systems, where an abundance of scales of interaction is likely to further unveil the complexity and stability of large scale networks or coordinative structures.

## Materials and methods

### Participants

120 participants (76 female, age 24±8 yrs.) participated in the experiment, making up 15 independent ensembles of eight. All participants were right-handed except 4, who were all able to complete the tasks without difficulty. The protocol was approved by Florida Atlantic University Institutional Review Board and in agreement with the Declaration of Helsinki. Written informed consent was obtained from all participants prior to the experiment.

### Experimental setup

**Fig 6:**
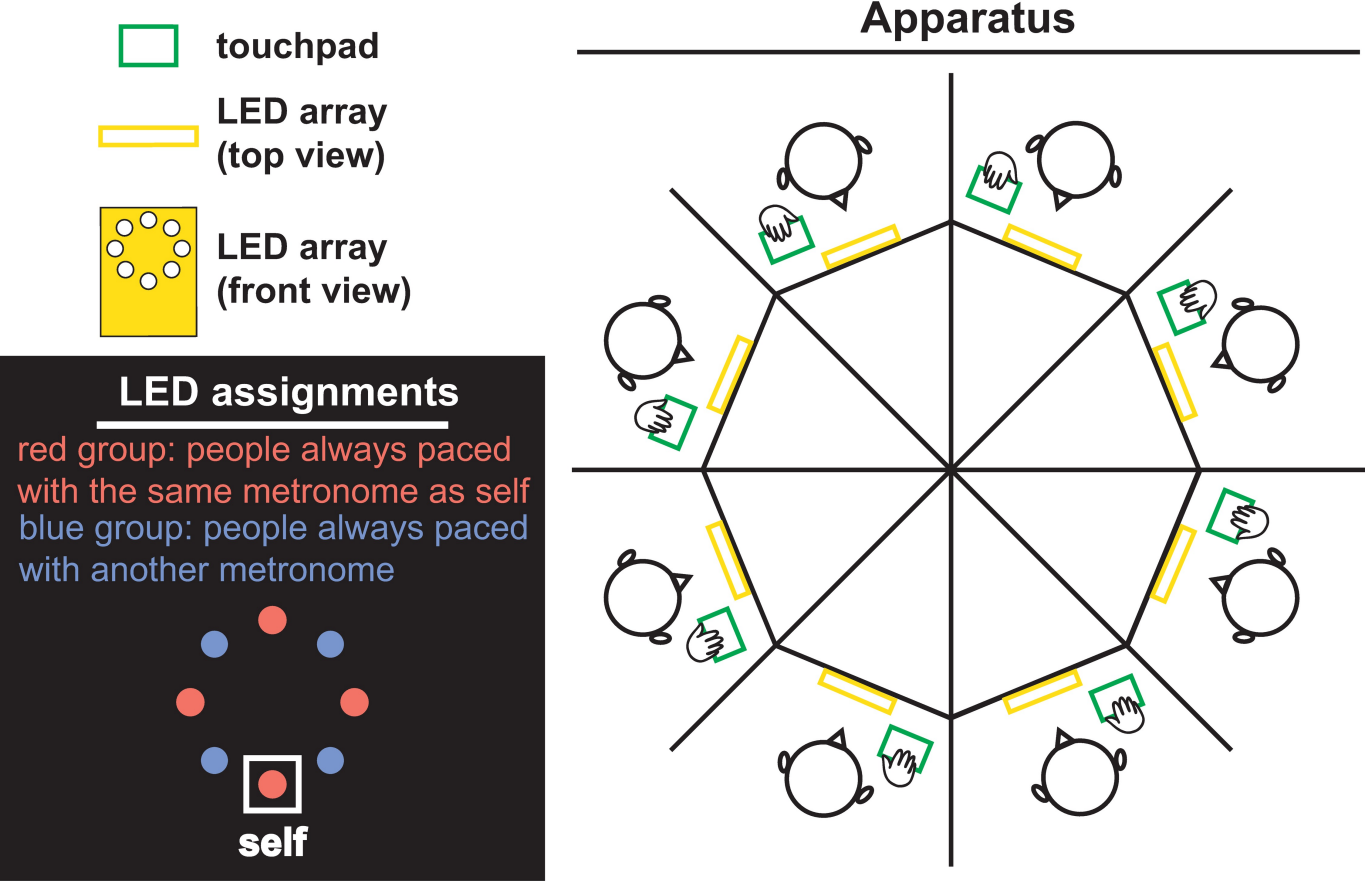
Experimental setup. Eight participants are seated around an octagonal table; they do not have direct vision of each other. Rather, they are exposed to each other’s tapping behavior through touchpads (record tapping; green) and arrays of LEDs (display self and others’ taps as flashes; yellow). On each LED array, there is a one-to-one correspondence between LEDs and participants. (black panel) The mapping was rotated for each array so that a participant always saw self-behavior at the lowest LED (white box). All LEDs labeled red represent people who were paced by metronomes of the same frequency as for self. LEDs labelled blue represent people paced to metronomes at another frequency (actual LEDs were all in the same color). By such metronome assignment, participants of the same ensemble were split into two initial frequency groups. By manipulating the metronome difference between the two groups, we created different levels of diversity, thereby inducing integration~segregation at different spatial scales.

For each ensemble of eight, participants were randomly seated in booths around an octagonal table. They did not have direct visual contact with each other. Each participant was equipped with a touchpad (green rectangle in Fig 6) and an array of eight light-emitting photodiodes (LEDs; yellow in Fig 6). Each tap of a participant was broadcast to all participants (including self) in real time as a single flash of an assigned LED (hand contacts touchpad, light on; hand leaves touchpad, light off). The tap~flash signals were converted and transmitted through a signal processing pipeline consisting of a PC flanked by two microcontrollers (MCs; one for input, one for output; communicates with the PC through serial port at 57600 bps). The input MC samples movement data from the touchpads at 250 Hz (1 = touch, 0 = leave) and sends data to the PC. Dedicated software (written in C++) runs on the PC, which receives tapping data from the input MC, and controls the spatial configuration of LEDs and the network connectivity among participants. The spatial configuration map assigns each LED on each array to represent a particular participant. The spatial configuration map was randomized across different ensembles of eight, but fixed for each ensemble throughout an experimental session. In this particular experiment, the network connectivity map determines whether a particular participant can see (1) only self-produced flashes; (2) self-produced flashes and a metronome (computer generated flashes, see **Procedures**); or (3) self- and other-produced flashes. After the spatial and network mapping are completed, the PC sends 64 bit data to 8 LED arrays via the output MC, synchronized to each sample from the input MC (tap-to-flash latency 2.5–4.5ms, less than 1% of the shortest period of metronomes).

### Procedures

Each trial of the experiment lasted 68s and consisted of three stages. In *Stage 1* (5s), participants tapped rhythmically at their own comfortable frequency, only seeing self-produced flashes (Fig 6, black inset “self”). In *Stage 2* (10s), all the non-self LEDs started to flash in synchrony at a preassigned frequency, basically a metronome (initial phase randomized). Participants were instructed to match their own tapping frequency to the metronome frequency, and remain tapping at that frequency throughout the rest of the trial even after the metronome disappeared. Following a 3s transient, subjects were exposed to each other’s rhythmic behavior (*Stage 3*, 50s), each LED flashed corresponding to a particular participant’s taps.

We manipulated intergroup behavior by assigning metronomes of different frequencies to different participants. In order to emphasize frequency diversity, spatial symmetry was imposed as follows: from each participant’s perspective, persons presented at the north, west, and east of the center of the LED array were always paced with the same metronome as self (south to center), whilst the others were paced with another metronome. Thus, metronome assignment was designed to split eight people into two initial frequency groups (red group and blue group in Fig 6, black inset). Diversity thus appears across groups not within groups. Specifically, for each trial, group metronomes were assigned following one of the three conditions: (1) 1.5 Hz vs. 1.5 Hz, (2) 1.65 Hz vs. 1.35 Hz, and (3) 1.8 Hz vs. 1.2 Hz. With the same mean frequency (1.5Hz), the three conditions correspond to three levels of between-group metronome difference (*δ f*) which we term a diversity parameter: *δ f* = 0 Hz, *δ f* = 0.3 Hz, *δ f* = 0.6 Hz.

Each ensemble of eight participants completed 24 trials in random order, including 6 trials in which participants were only connected to people within their own group (results not reported in this paper) and 18 trials in which every participant was connected to every other participant. In the present paper, we consider the effect of different levels of between-group difference on fully connected ensembles of eight people.

### Statistical analyses

Distributions of relative phase (*ϕ*) and frequency ratio (*FR*) were compared to chance level using permutation tests. Ten thousand randomly permuted time series were used for constructing the confidence intervals of chance level distributions. The significance level was chosen to be $\hat{p}$ = 0.05 (with Bonferroni correction). Computational details are shown in Section A and Section D in S1 File.

To compare the level of phase-locking in different conditions, two-way ANOVA was used (2×3 for relation × *δ f*) with Type III Sums of Squares; Tukey Honest Significant Difference tests were used for post hoc comparisons (see Section B in S1 File for details).

To measure the level of integration between groups, we regressed the level of within-group phase locking against between group phase-locking separately for 3 diversity levels. The slopes of the regression lines (*β*_*1*_^*δ f*^) reflect the level of integration (positive slope = integration, negative slope = segregation). The critical level of diversity (*δ f* ^*^), corresponding to zero-slope (*β*_*1*_ = 0), was found through linear interpolation (see Section C in S1 File for details).

## Supporting information

S1 File Supporting information. Re: “Critical diversity: divided or united states of social coordination” by Zhang et al.
(PDF)

## References

[1] Haken H. Synergetics: Introduction and Advanced Topics. Berlin, Heidelberg: Springer Berlin Heidelberg; 2004 doi: 10.1007/978-3-662-10184-1

[2] Camazine S, Deneubourg J-L, Franks NR, Sneyd J, Theraulaz G, Bonabeau E. Self-Organization in Biological Systems. Princeton University Press; 2003.

[3] Kelso JAS. Dynamic Patterns: The Self-Organization of Brain and Behavior. Cambridge, Massachusetts: The MIT Press; 1995.

[4] Miller JH, Page SE. Complex Adaptive Systems: an Introduction to Computational Models of Social Life. Princeton university press; 2009.

[5] Brown FA. Living clocks: the clocks are accounted for as “open systems” depending upon subtle geophysical rhythms. Science. 1959;130: 1535–1544. doi: 10.1126/science.130.3388.1535 13804924

[6] Wunderlich FM. Symphonies of urban places: urban rhythms as traces of time in space a study of “urban rhythms.” Place Locat Stud Environ Aesthet Semiot VI. 2008; 91–111.

[7] Glass L. Synchronization and rhythmic processes in physiology. Nature. 2001;410: 277–284. doi: 10.1038/35065745 11258383 10.1038/35065745

[8] Buzsáki G. Rhythms of the Brain. Oxford University Press; 2006 doi: 10.1093/acprof:oso/9780195301069.001.0001

[9] Blasius B, Huppert A, Stone L. Complex dynamics and phase synchronization in spatially extended ecological systems. Nature. 1999;399: 354–9. doi: 10.1038/20676 10360572 10.1038/20676

[10] Repp BH. Sensorimotor synchronization: a review of the tapping literature. Psychon Bull Rev. 2005;12: 969–992. doi: 10.3758/BF03206433 16615317 10.3758/bf03206433

[11] Kelso JAS, Del Colle JD, Schöner G. Action-perception as a pattern formation process Attention and performance 13: Motor representation and control. Hillsdale, NJ, US: Lawrence Erlbaum Associates, Inc; 1990 pp. 139–169.

[12] Kelso JAS. Phase transitions and critical behaviour in human bimanual coordination. Am J Physiol Regul Integr Comp Physiol. 1984;246: R1000–1004. 10.1152/ajpregu.1984.246.6.R1000 6742155

[13] Schöner G, Kelso JAS. Dynamic pattern generation in behavioral and neural systems. Science. 1988;239: 1513–1520. doi: 10.1126/science.3281253 3281253 10.1126/science.3281253

[14] Assisi CG, Jirsa VK, Kelso JAS. Dynamics of multifrequency coordination using parametric driving: theory and experiment. Biol Cybern. 2005;93: 6–21. doi: 10.1007/s00422-005-0558-y 15926066 10.1007/s00422-005-0558-y

[15] Peper CE, Beek PJ, van Wieringen PCW. Multifrequency coordination in bimanual tapping: Asymmetrical coupling and signs of supercriticality. J Exp Psychol Hum Percept Perform. 1995;21: 1117–1138. doi: 10.1037/0096-1523.21.5.1117

[16] Oullier O, de Guzman GC, Jantzen KJ, Lagarde J, Kelso JAS. Social coordination dynamics: measuring human bonding. Soc Neurosci. 2008;3: 178–192. doi: 10.1080/17470910701563392 18552971 10.1080/17470910701563392 PMC2156197

[17] Richardson MJ, Marsh KL, Isenhower RW, Goodman JRL, Schmidt RC. Rocking together: dynamics of intentional and unintentional interpersonal coordination. Hum Mov Sci. 2007;26: 867–891. doi: 10.1016/j.humov.2007.07.002 17765345 10.1016/j.humov.2007.07.002

[18] Tognoli E, Lagarde J, de Guzman GC, Kelso JAS. The phi complex as a neuromarker of human social coordination. Proc Natl Acad Sci. 2007;104: 8190–8195. doi: 10.1073/pnas.0611453104 17470821 10.1073/pnas.0611453104 PMC1859993

[19] Dumas G, Nadel J, Soussignan R, Martinerie J, Garnero L. Inter-brain synchronization during social interaction. Lauwereyns J, editor. PLoS One. 2010;5: e12166 doi: 10.1371/journal.pone.0012166 20808907 10.1371/journal.pone.0012166 PMC2923151

[20] Noy L, Dekel E, Alon U. The mirror game as a paradigm for studying the dynamics of two people improvising motion together. Proc Natl Acad Sci. 2011;108: 20947–20952. doi: 10.1073/pnas.1108155108 22160696 10.1073/pnas.1108155108 PMC3248496

[21] Hasson U, Ghazanfar AA, Galantucci B, Garrod S, Keysers C. Brain-to-brain coupling: a mechanism for creating and sharing a social world. Trends Cogn Sci. 2012;16: 114–121. doi: 10.1016/j.tics.2011.12.007 22221820 10.1016/j.tics.2011.12.007 PMC3269540

[22] Schmidt RC, Carello C, Turvey MT. Phase transitions and critical fluctuations in the visual coordination of rhythmical movements between people. J Exp Psychol Hum Percept Perform. 1990;16: 227–247. 2142196 10.1037//0096-1523.16.2.227

[23] Varela F, Lachaux JP, Rodriguez E, Martinerie J. The brainweb: phase synchronization and large-scale integration. Nat Rev Neurosci. 2001;2: 229–239. doi: 10.1038/35067550 11283746 10.1038/35067550

[24] Tognoli E, Kelso JAS. Brain coordination dynamics: true and false faces of phase synchrony and metastability. Prog Neurobiol. 2009;87: 31–40. doi: 10.1016/j.pneurobio.2008.09.014 18938209 10.1016/j.pneurobio.2008.09.014 PMC3020160

[25] Kelso JAS, de Guzman GC, Reveley C, Tognoli E. Virtual Partner Interaction (VPI): exploring novel behaviors via coordination dynamics. Sporns O, editor. PLoS One. 2009;4: e5749 doi: 10.1371/journal.pone.0005749 19492044 10.1371/journal.pone.0005749 PMC2685001

[26] Dumas G, de Guzman GC, Tognoli E, Kelso JAS. The human dynamic clamp as a paradigm for social interaction. Proc Natl Acad Sci. 2014;111: E3726–E3734. doi: 10.1073/pnas.1407486111 25114256 10.1073/pnas.1407486111 PMC4156776

[27] Kostrubiec V, Dumas G, Zanone P-G, Kelso JAS. The Virtual Teacher (VT) paradigm: learning new patterns of interpersonal coordination using the Human Dynamic Clamp. Marinazzo D, editor. PLoS One. 2015;10: e0142029 doi: 10.1371/journal.pone.0142029 26569608 10.1371/journal.pone.0142029 PMC4646495

[28] Lagarde J, Peham C, Licka T, Kelso JAS. Coordination dynamics of the horse-rider system. J Mot Behav. 2005;37: 418–424. doi: 10.3200/JMBR.37.6.418-424 16280312 10.3200/JMBR.37.6.418-424 PMC1821095

[29] Pfau T, Spence A, Starke S, Ferrari M, Wilson A. Modern riding style improves horse racing times. Science. 2009;325: 289–289. doi: 10.1126/science.1174605 19608909 10.1126/science.1174605

[30] Haken H, Kelso JAS, Bunz H. A theoretical model of phase transitions in human hand movements. Biol Cybern. 1985;51: 347–356. doi: 10.1007/BF00336922 3978150 10.1007/BF00336922

[31] Schöner G, Kelso JAS. A dynamic pattern theory of behavioral change. J Theor Biol. 1988;135: 501–524. doi: 10.1016/S0022-5193(88)80273-X

[32] Fuchs A, Jirsa VK, Haken H, Kelso JAS. Extending the HKB model of coordinated movement to oscillators with different eigenfrequencies. Biol Cybern. 1996;74: 21–30. doi: 10.1007/BF00199134 8573650 10.1007/BF00199134

[33] Schöner G, Haken H, Kelso JAS. A stochastic theory of phase transitions in human hand movement. Biol Cybern. 1986;53: 247–257. doi: 10.1007/BF00336995 3955100 10.1007/BF00336995

[34] Schöner G, Jiang WY, Kelso JAS. A synergetic theroy of quadrupedal gaits and gait transistions. J Theor Biol. 1990;142: 359–391. 2338828 10.1016/s0022-5193(05)80558-2

[35] Collins JJ, Stewart I. Coupled nonlinear oscillators and the symmetries of animal gaits. J Nonlinear Sci. 1993;3: 349–392. doi: 10.1007/BF02429870

[36] Jeka JJ, Kelso JAS. Manipulating symmetry in the coordination dynamics of human movement. J Exp Psychol Hum Percept Perform. 1995;21: 360–374. 7714477 10.1037//0096-1523.21.2.360

[37] Golubitsky M, Stewart I, Buono P-LL, Collins JJ. Symmetry in locomotor central pattern generators and animal gaits. Nature. 1999;401: 693–695. doi: 10.1038/44416 10537106 10.1038/44416

[38] Kelso JAS, Jeka JJ. Symmetry breaking dynamics of human multilimb coordination. J Exp Psychol Hum Percept Perform. 1992;18: 645–668. doi: 10.1037/0096-1523.18.3.645 1500867 10.1037//0096-1523.18.3.645

[39] Buck J, Buck E. Synchronous fireflies. Sci Am. 1976;234: 74–85. doi: 10.1038/scientificamerican0576-74 1273569 10.1038/scientificamerican0576-74

[40] Edelman GM, Tononi GS. Reentry and the dynamic core: neural correlates of conscious experience In: Metzinger T, editor. Neural Correlates of Consciousness. MIT Press; 2000.

[41] Néda Z, Ravasz E, Brechet Y, Vicsek T, Barabási A-L. The sound of many hands clapping. Nature. 2000;403: 849–850. doi: 10.1038/35002660 10706271 10.1038/35002660

[42] Strogatz SH. From Kuramoto to Crawford: exploring the onset of synchronization in populations of coupled oscillators. Phys D Nonlinear Phenom. 2000;143: 1–20. doi: 10.1016/S0167-2789(00)00094-4

[43] Kuramoto Y. Chemical Oscillations, Waves and Turbulence. [Internet]. Berlin: Springer; 1983 doi: 10.1007/978-3-642-12601-7

[44] Néda Z, Ravasz E, Vicsek T, Brechet Y, Barabási A-L. Physics of the rhythmic applause. Phys Rev E—Stat Physics, Plasmas, Fluids, Relat Interdiscip Top. 2000;61: 6987–6992. doi: 10.1103/PhysRevE.61.6987 10.1103/physreve.61.6987 11088392

[45] Mirollo RE, Strogatz SH. Synchronization of pulse-coupled biological oscillators. SIAM J Appl Math. 1990;50: 1645–1662.

[46] Richardson MJ, Garcia RL, Frank TD, Gergor M, Marsh KL. Measuring group synchrony: a cluster-phase method for analyzing multivariate movement time-series. Front Physiol. 2012;3: 1–10. doi: 10.3389/fphys.2012.00001 23091463 10.3389/fphys.2012.00405 PMC3475977

[47] Fuchs A. Nonlinear Dynamics in Complex Systems. Berlin, Heidelberg: Springer Berlin Heidelberg; 2013 doi: 10.1007/978-3-642-33552-5

[48] Kelso JAS. Multistability and metastability: understanding dynamic coordination in the brain. Philos Trans R Soc B Biol Sci. 2012;367: 906–918. doi: 10.1098/rstb.2011.0351 22371613 10.1098/rstb.2011.0351 PMC3282307

[49] Von Holst E. On the nature of order in the central nervous system The Collected Papers of Erich von Holst Vol 1, The Behavioral Physiology of Animal and Man. Coral Gables, FL: University of Miami Press; 1973 pp. 3–32.

[50] Tognoli E, Kelso JAS. The metastable brain. Neuron. Elsevier Inc.; 2014;81: 35–48. doi: 10.1016/j.neuron.2013.12.022 24411730 10.1016/j.neuron.2013.12.022 PMC3997258

[51] Kelso JAS. The dynamic brain in action: coordinative structures, criticality, and coordination dynamics. In: Plenz D, Niebur E, editors. Criticality in Neural Systems. 2014. pp. 67–104. doi: 10.1002/9783527651009.ch4

[52] de Guzman GC, Kelso JAS. Multifrequency behavioral patterns and the phase attractive circle map. Biol Cybern. 1991;64: 485–495. doi: 10.1007/BF00202613 1863660 10.1007/BF00202613

[53] Kelso JAS, de Guzman GC. Order in time: how the cooperation between the hands informs the design of the brain In: Haken H, editor. Neural and Synergetic Computers. Berlin, Heidelberg: Springer Berlin Heidelberg; 1988 pp. 180–196. doi: 10.1007/978-3-642-74119-7_13

[54] Strogatz SH, Goldenfeld N. Sync: The emerging science of spontaneous order. Phys Today. 2004;57: 59–60. doi: 10.1063/1.1784276

[55] Buck J. Synchronous rhythmic flashing of fireflies. II. Q Rev Biol. 1988;63: 265–289. doi: 10.1086/415929 3059390 10.1086/415929

[56] Strogatz SH, Stewart I. Coupled oscillators and biological synchronization. Sci Am. 1993;269: 102–109. doi: 10.1038/scientificamerican1293-102 8266056 10.1038/scientificamerican1293-102

[57] Winfree AT. The Geometry of Biological Time [Internet]. Springer New York, NY: Springer New York; 2001 doi: 10.1007/978-1-4757-3484-3

[58] Buzsáki G. Theta rhythm of navigation: link between path integration and landmark navigation, episodic and semantic memory. Hippocampus. 2005;15: 827–840. doi: 10.1002/hipo.20113 16149082 10.1002/hipo.20113

[59] Alderisio F, Bardy BG, di Bernardo M. Entrainment and synchronization in networks of Rayleigh–van der Pol oscillators with diffusive and Haken–Kelso–Bunz couplings. Biol Cybern. Springer Berlin Heidelberg; 2016;110: 151–169. doi: 10.1007/s00422-016-0685-7 27108135 10.1007/s00422-016-0685-7 PMC4903116

[60] Nishikawa T, Motter AE. Symmetric states requiring system asymmetry. Phys Rev Lett. 2016;117: 1–5. doi: 10.1103/PhysRevLett.117.114101 10.1103/PhysRevLett.117.114101 27661690

[61] Valdesolo P, DeSteno D. Synchrony and the social tuning of compassion. Emotion. 2011;11: 262–266. doi: 10.1037/a0021302 21500895 10.1037/a0021302

[62] Hove MJ, Risen JL. It’s all in the timing: interpersonal synchrony increases affiliation. Soc Cogn. 2009;27: 949–960. doi: 10.1521/soco.2009.27.6.949

[63] Zhang M, Dumas G, Kelso JAS, Tognoli E. Enhanced emotional responses during social coordination with a virtual partner. Int J Psychophysiol. 2016;104: 33–43. doi: 10.1016/j.ijpsycho.2016.04.001 27094374 10.1016/j.ijpsycho.2016.04.001 PMC4899205

[64] Fogel A, Nwokah E, Dedo JY, Messinger D. Social process theory of emotion: A dynamic systems approach. Soc Dev. 1992;1: 122–142. doi: 10.1111/j.1467-9507.1992.tb00116.x

[65] Wheatley T, Kang O, Parkinson C, Looser CE. From mind perception to mental connection: synchrony as a mechanism for social understanding. Soc Personal Psychol Compass. 2012;6: 589–606. doi: 10.1111/j.1751-9004.2012.00450.x

[66] Oullier O, Kelso JAS. Social coordination, from the perspective of Coordination Dynamics In: Meyers RA, editor. Encyclopedia of Complexity and Systems Science. New York, NY: Springer New York; 2009 pp. 8198–8213. doi: 10.1007/978-0-387-30440-3_486

[67] Schmidt RC, Fitzpatrick P, Caron R, Mergeche J. Understanding social motor coordination. Hum Mov Sci. Elsevier B.V.; 2011;30: 834–845. doi: 10.1016/j.humov.2010.05.014 20817320 10.1016/j.humov.2010.05.014

[68] Sekara V, Stopczynski A, Lehmann S. Fundamental structures of dynamic social networks. Proc Natl Acad Sci. 2016;113: 9977–9982. doi: 10.1073/pnas.1602803113 27555584 10.1073/pnas.1602803113 PMC5018769

[69] Boiger M, Mesquita B. The construction of emotion in interactions, relationships, and cultures. Emot Rev. 2012;4: 221–229. doi: 10.1177/1754073912439765

[70] Carneiro RR. On the relationship between size of population and complexity of social organization. Southwest J Anthropol. 1967;23: 234–243. Available: http://www.jstor.org/stable/3629251

[71] Changizi MA, He D. Four correlates of complex behavioral networks: differentiation, behavior, connectivity, and compartmentalization: Carving networks at their joints. Complexity. 2005;10: 13–40. doi: 10.1002/cplx.20085

[72] Weng G, Bhalla US, Iyengar R. Complexity in biological signaling systems. Science. 1999;284: 92–96. doi: 10.1126/science.284.5411.92 10102825 10.1126/science.284.5411.92 PMC3773983

[73] Stouffer DB, Bascompte J. Compartmentalization increases food-web persistence. Proc Natl Acad Sci. 2011;108: 3648–3652. doi: 10.1073/pnas.1014353108 21307311 10.1073/pnas.1014353108 PMC3048152

[74] Kirschner M, Gerhart J. Evolvability. Proc Natl Acad Sci. 1998;95: 8420–8427. doi:VL—95 9671692 10.1073/pnas.95.15.8420 PMC33871

[75] Ash J, Newth D. Optimizing complex networks for resilience against cascading failure. Phys A Stat Mech its Appl. 2007;380: 673–683. doi: 10.1016/j.physa.2006.12.058

[76] Edelman GM, Gally J. Degeneracy and complexity in biological systems. Proc Natl Acad Sci. 2001;98: 13763–13768. doi: 10.1073/pnas.231499798 11698650 10.1073/pnas.231499798 PMC61115

[77] Bar-Yam Y. Multiscale variety in complex systems. Complexity. 2004;9: 37–45. doi: 10.1002/cplx.20014

[78] Sporns O. Network attributes for segregation and integration in the human brain. Curr Opin Neurobiol. Elsevier Ltd; 2013;23: 162–171. doi: 10.1016/j.conb.2012.11.015 23294553 10.1016/j.conb.2012.11.015

[79] Tononi GS, Sporns O, Edelman GM. A measure for brain complexity: relating functional segregation and integration in the nervous system. Proc Natl Acad Sci. 1994;91: 5033–5037. doi: 10.1073/pnas.91.11.5033 8197179 10.1073/pnas.91.11.5033 PMC43925

[80] McPherson M, Smith-Lovin L, Cook JM. Birds of a feather: homophily in social networks. Annu Rev Sociol. 2001;27: 415–444. doi: 10.1146/annurev.soc.27.1.415

[81] Moody J. Race, school integration, and friendship segregation in America. Am J Sociol. 2001;107: 679–716. doi: 10.1086/338954

[82] Schelling TC. Dynamic models of segregation. J Math Sociol. 1971;1: 143–186. doi: 10.1080/0022250X.1971.9989794

[83] Stark TH, Flache A. The double edge of common interest. Sociol Educ. 2012;85: 179–199. doi: 10.1177/0038040711427314

[84] Blau PM. Inequality and Heterogeneity: a Primitive Theory of Social Structure. New York, NY: Free Press; 1977.

[85] Kelso JAS, Engstrom DA. The Complementary Nature. MIT press; 2006.

[86] Kelso JAS. Instabilities and phase transitions in human brain and behavior. Front Hum Neurosci. 2010;4: 23 doi: 10.3389/fnhum.2010.00023 20461234 10.3389/fnhum.2010.00023 PMC2866541

[87] Scheffer M, Bascompte J, Brock WA, Brovkin V, Carpenter SR, Dakos V, et al Early-warning signals for critical transitions. Nature. 2009;461: 53–59. doi: 10.1038/nature08227 19727193 10.1038/nature08227

[88] Wilson KG. Problems in physics with many scales of length. Sci Am. 1979;241: 158–179. doi: 10.1038/scientificamerican0879-158

[89] Simon HA. The organization of complex systems. Hierarchy theory: The challenge of complex systems. 1977 pp. 245–261. doi: 10.1007/978-94-010-9521-1_14

[90] Schelling TC. Micromotives and Macrobehavior. 1st ed. New York: Norton; 1978.

[91] Leibold MA, Holyoak M, Mouquet N, Amarasekare P, Chase JM, Hoopes MF, et al The metacommunity concept: a framework for multi-scale community ecology. Ecol Lett. 2004;7: 601–613. doi: 10.1111/j.1461-0248.2004.00608.x

[92] Parra J, Kalitzin SN, Iriarte J, Blanes W, Velis DN, Lopes da Silva FH. Gamma-band phase clustering and photosensitivity: Is there an underlying mechanism common to photosensitive epilepsy and visual perception? Brain. 2003;126: 1164–1172. doi: 10.1093/brain/awg109 12690055 10.1093/brain/awg109

[93] Tognoli E, Kelso JAS. Enlarging the scope: grasping brain complexity. Front Syst Neurosci. 2014;8: 122 doi: 10.3389/fnsys.2014.00122 25009476 10.3389/fnsys.2014.00122 PMC4070173

[94] Kelso JAS, Dumas G, Tognoli E. Outline of a general theory of behavior and brain coordination. Neural Networks. Elsevier Ltd; 2013;37: 120–131. doi: 10.1016/j.neunet.2012.09.003 23084845 10.1016/j.neunet.2012.09.003 PMC3914303

[95] Takamatsu A, Tanaka R, Fujii T. Hidden symmetry in chains of biological coupled oscillators. Phys Rev Lett. 2004;92: 228102–1. doi: 10.1103/PhysRevLett.92.228102 15245261 10.1103/PhysRevLett.92.228102

[96] Yokoyama K, Yamamoto Y. Three people can synchronize as coupled oscillators during sports activities. Diedrichsen J, editor. PLoS Comput Biol. 2011;7: e1002181 doi: 10.1371/journal.pcbi.1002181 21998570 10.1371/journal.pcbi.1002181 PMC3188505

[97] Kuramoto Y, Battogtokh D. Coexistence of coherence and incoherence in nonlocally coupled phase oscillators. Nonlinear Phenom Complex Syst. 2002;5: 380–385. Available: http://www.j-npcs.org/abstracts/vol2002/v5no4/v5no4p380.html

[98] Holling CS. Understanding the complexity of economic, ecological, and social systems. Ecosystems. 2001;4: 390–405. doi: 10.1007/s10021-00-0101-5

[99] Kelso JAS. An essay on understanding the mind. Ecol Psychol. 2008;20: 180–208. doi: 10.1080/10407410801949297 19865611 10.1080/10407410801949297 PMC2768408

[100] Palla G, Barabási A-L, Vicsek T. Quantifying social group evolution. Nature. 2007;446: 664–667. doi: 10.1038/nature05670 17410175 10.1038/nature05670

[101] Wang J, Suri S, Watts DJ. Cooperation and assortativity with dynamic partner updating. Proc Natl Acad Sci. 2012;109: 14363–14368. doi: 10.1073/pnas.1120867109 22904193 10.1073/pnas.1120867109 PMC3437882

[102] Haken H, Peper CE, Beek PJ, Daffertshofer A. A model for phase transitions in human hand movements during multifrequency tapping. Phys D Nonlinear Phenom. 1996;90: 179–196. doi: 10.1016/0167-2789(95)00235-9

[103] Arnold VI. Small denominators. I. Mapping of the circumference onto itself. Collected Works. Berlin, Heidelberg: Springer Berlin Heidelberg; 2009 pp. 152–223. doi: 10.1007/978-3-642-01742-1_10

[104] Jensen O, Colgin LL. Cross-frequency coupling between neuronal oscillations. Trends Cogn Sci. 2007;11: 267–269. doi: 10.1016/j.tics.2007.05.003 17548233 10.1016/j.tics.2007.05.003

[105] Lemke JL. Across the scales of time: artifacts, activities, and meanings in ecosocial systems. Mind, Cult Act. 2000;7: 273–290. doi: 10.1207/S15327884MCA0704_03

