# Supplementary material for: Critical diversity: Divided or united states of social coordination
Source: PLoS One. 2018 Apr 4;13(4):e0193843. doi: 10.1371/journal.pone.0193843 (PMC5884498; doi:10.1371/journal.pone.0193843)
Supplement: S1 File — Re: “Critical diversity: divided or united states of social coordination” by Zhang et al. (PDF) [file pone.0193843.s001.pdf]

S1. Supporting information

Re: “Critical diversity: divided or united states of social coordination” by Zhang et al.

Section A-Section D detail the main data processing techniques and statistical methods involved in the main text. Section A describes the computation of phase, relative phase (ϕ), phase-locking value (PLV), instantaneous frequency and frequency ratio (FR). Section B shows the statistical method for comparing the level of phase-locking (Fig 4A). Section C explains how linear regression was used to assess the level of integration and how to compute critical diversity (Fig 4B). Section D describes how to construct the confidence interval for null distributions in Fig E (corresponding to Fig 2) and Fig 3.

Section E-Section L show supplementary results to those reported in the main text. Section E shows that participants were able to tap at the required frequency according to instructions. Section F shows how episodes of strong interaction were extracted from relative phase time series, and shows distributions of the duration and phase patterns in those episodes. Section G shows statistical comparisons of the relative phase distributions in Fig 2 to chance level distributions. Section H shows statistically how dyadic phase relations can be influenced by chances in its surrounding network structure. Section I provides additional discussion on metastable dynamics in multiagent coordination complementing Fig 1 and Fig 5. Section J presents a method to quantify metastability (metastable index), the validation of the method with simulated data, and results obtained from the present experimental data. Section K shows how simulated data used in Section J were obtained. Section L shows the main effects corresponding to the interaction effects shown in Fig 4A.

Section A. Preprocessing of recorded signals

In the current study, we characterize social coordination in terms of frequency and phase relations. Here we detail how frequency and phase related variables were transformed from raw signals (square waves consisting of zeros and ones, see Experimental Setup). We define the inter-tap interval (ITI) as the time

difference between the onsets of two consecutive taps. Instantaneous Frequency (F) is the reciprocal of ITI, interpolated linearly between taps in accord with the original sampling rate (250Hz). We obtained Phase (θ_i) by first assigning the value $2\pi(n-1)$ to the onset of the nth tap of the ith individual, and then interpolating samples in between with a cubic spline method. Further, we define relational variables Frequency Ratio (FR_{ij}) and Relative Phase (ϕ_{ij}) between individual i and j as

$$FR_{ij} = \frac{\min(F_i, F_j)}{\max(F_i, F_j)}$$

and

$$\phi_{ij} = \theta_i - \theta_j$$

respectively.

To quantify the degree of phase coordination, we segment each time series into consecutive 3s windows, and calculate the Phase-Locking Value (PLV) within such windows.

$$PLV = 1 - CV = \frac{1}{N}\left|\sum_{n=1}^{N} e^{i\phi[n]}\right|$$

where CV is circular variance, and N is the total number of samples in a window (750 pts). PLV ranges from zero to one. A value of one indicates the maximal degree of coordination, and a value of zero indicates no coordination.

Section B. Multivariate analysis of variance (MANOVA)

To study how dyadic coordination within and between initial groups (denoted as variable “relation” with two categories: “within-group” and “between-group”) changes as diversity varies (i.e. between-group difference in frequency predispositions, δf=0, 0.3, 0.6 Hz), we performed a 2×3 (relation × δf) MANOVA to compare the mean PLV in different conditions, using Type III Sums of Squares. Multiple comparisons were performed using Tukey HSD (honest significant difference) tests.

Section C. Linear regressions and critical frequency identification

To study the macro organization of groups, we examined the relation between within-group and between-group phase coordination, and how it changes as diversity (δf) increases. Least Square method was used to obtain the regression line for each δf,

$$PLV_{between-group}^{\delta f} = \beta_0^{\delta f} + \beta_1^{\delta f}\ PLV_{within-group}^{\delta f} + \epsilon^{\delta f}$$

The slope $\beta_1 = \beta_1^{\delta f}$ is the relation between within- and between-group coordination, an index of the degree of integration between two initial groups. If $\beta_1 = 1$, there is only one undifferentiated supergroup. $0 < \beta_1 < 1$ indicates that initial groups integrated into a supergroup but there is remnant of the diversity (coordination with one group increases the coordination with the other group but not as much as when there is no diversity). $\beta_1 < 0$ indicates that initial groups remain segregated (coordination with one group decreases coordination with another group).

If there exists a diversity *δf* such that within-group coordination does not vary with between-group coordination (degree of integration $\beta_1^{\delta f} = 0$), we call it a critical diversity (δf^*) - a separatrix between regimes of integration and segregation of two initial groups. To obtain δf^*, we regressed the degree of integration β_1 against diversity *δf*,

$$\beta_1 = \alpha_0 + \alpha_1 \delta f + \varepsilon$$

and estimated where the regression line crosses $\beta_1 = 0$,

$$\delta f^* := -\frac{\alpha_0}{\alpha_1}$$

.

Section D. Distributional comparison

To verify how much the distribution of relational variables (FR, ϕ) in each condition reflects genuine coordination, we constructed chance level distributions by random permutations of all taps within each condition (i.e. taps produced following the same metronome). A total of 10,000 random permutations were performed. For each permutation, relational variables were computed following the same procedures as that of the original data (see Section A) and a probability density function (PDF) was computed for each variable using a 100-bin histogram. Given a significance level of p=0.0005 for each bin (based on Bonferroni correction for $\hat{p}$ =0.05 for the entire distribution), we computed the confidence interval around chance level distribution (two-tailed) as between $(100 - 50p)$ percentile and $50p$ percentile of the 10,000 random distributions for each bin. The real distribution is significantly different from chance at a specific value of FR or ϕ, if the probability density of this value is outside the confidence interval (seen as light-colored bands in main text Figs).

Section E. Participants tapped at (near) metronome frequencies as per instruction

Overall, participants followed instructions to tap at the metronome frequencies both during pacing (Fig A, top) and interaction (Fig A, bottom). On average, participants tapped a little faster than the metronome: by 0.074 ± 0.41Hz during pacing ($t(2070)=8.26$, $p<0.001$) and 0.14 ±0.45 Hz during interaction ($t(2071)=14.35$, $p<0.001$).

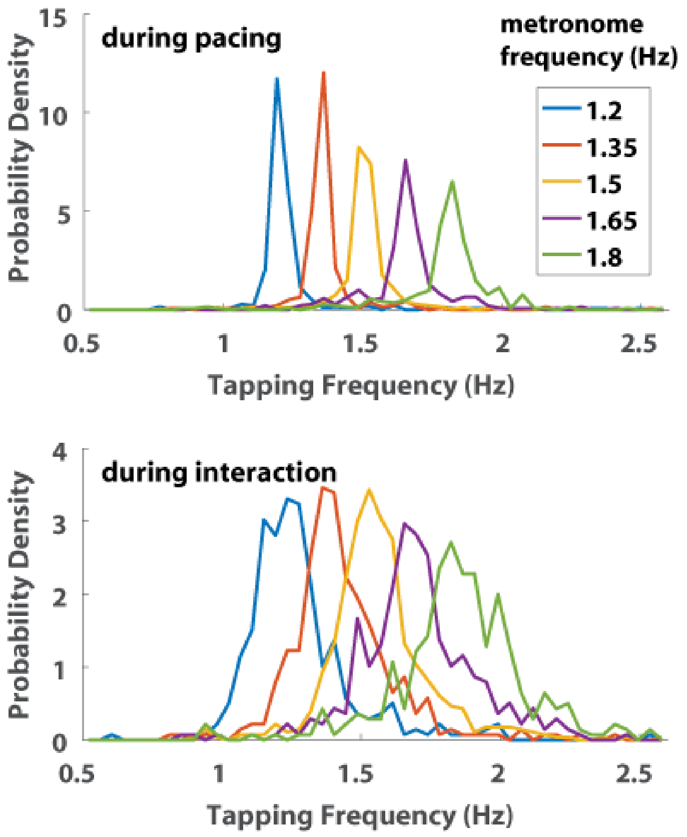

Fig A. Distributions of tapping frequency during pacing (top panel) and during interaction (bottom panel). Five Probability density functions of participants' tapping frequency are shown for five different metronome frequencies (1.5 Hz for δ f=0 Hz, 1.35 and 1.65Hz for δ f=0.3 Hz, 1.2 and 1.8Hz for δ f=0.6 Hz).

Section F. Episodes of strong phase coordination

To identify strongly attractive phase patterns and their persistence, an expert trained in coordination dynamics manually extracted the onsets and offsets of strong phase coordination (i.e. periods of constant phase relations with small fluctuation) in time series of dyadic relative phase. One relative phase time series was presented at a time and in random order. The inspector was blind from phase relations other than the one under inspection and the condition (i.e. δf) from which a time series was extracted.

The distribution of the duration of manually extracted episodes of strong coordination (Fig) shows that phase coordination primarily occurred for short periods (<10s); long lasting ones (stable phase locking) also appeared, but the occurrence was very rare.

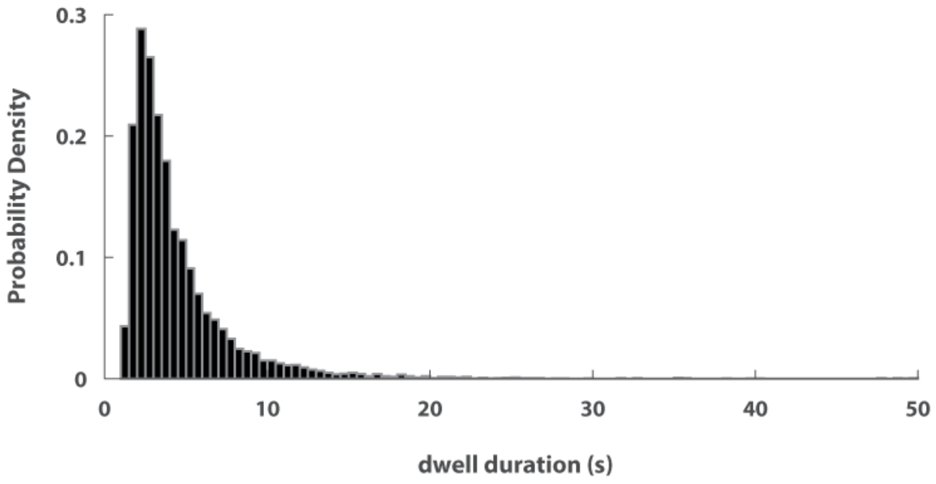

Fig B. Distribution of the durations of strong phase coordination.

Fig C shows the distributions of relative phase within those episodes of strong interaction. Within groups (Fig C(A)), both inphase (peak near ϕ $=0$) and antiphase (smaller peak near $\phi=\pm\pi$) are dominant phase patterns with inphase stronger than antiphase, regardless of diversity conditions (color). Between groups (Fig C(B)), the dominance of inphase pattern was retained across different levels of diversity, but antiphase gradually lost its attraction with increasing diversity (gradually flattened peaks near $\phi=\pm\pi$ from blue to red to yellow).

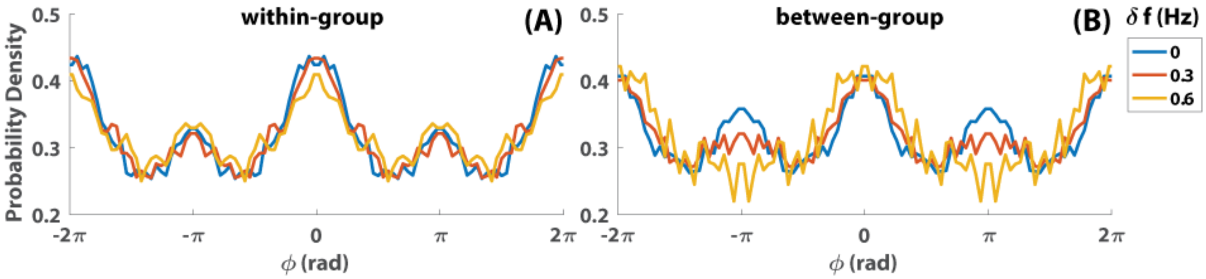

Fig C. Relative phase distribution during strong coordination for within-group dyads (A) and between-group dyads (B).

To see whether the relative phase relation was affected by the number of partners one simultaneously coordinated to, we computed for each agent the number of people he/she simultaneously engaged in strong interaction with (i.e. number of connections, the horizontal axis in Fig D) and the corresponding relative phase patterns adopted. We were particularly interested in the dominance of near inphase (i.e. $|\phi| < \pi/3$) and near antiphase ($|\phi| > 2\pi/3$) patterns (Fig D, blue, red respectively), for which we computed the probability density for each fixed number of connections (1 to 6; the maximal number connections one can make is 7, but its very rare occurrence made for an unreliable estimate, thus not shown). We found that the dominance of near inphase patterns gradually increased as one connected to more individuals; in contrast, the dominance of near antiphase patterns was steady across a small number of connections ($N \leqslant 4$) then gradually declined for a higher number of connections. This finding suggests that inphase, as the most symmetric form of phase pattern, may serve as a scaffold for individuals to simultaneously coordinate with multiple people (as in a rowing eight, for instance).

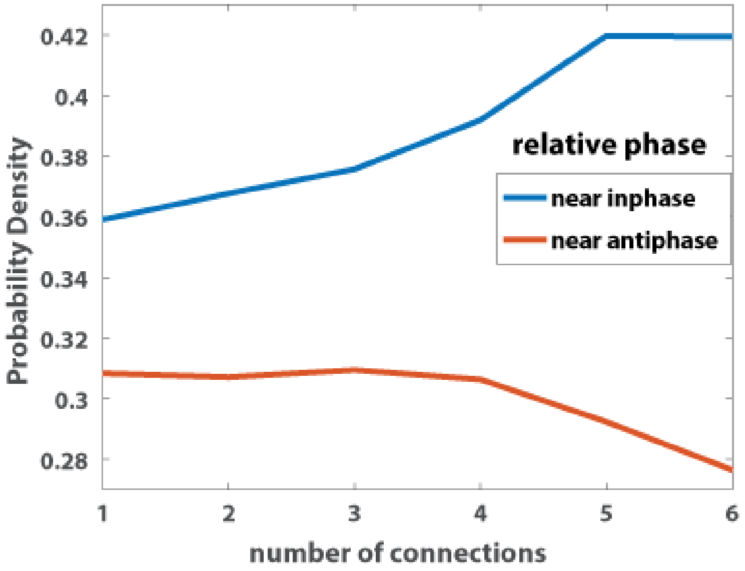

Fig D. Dominance of near-inphase vs. near-antiphase patterns as a function of the number of connections. Blue line indicates the probability density of near inphase patterns (|ϕ|< π/3) when participants simultaneously coordinated with different numbers of people (i.e. number of connections); red line indicates near-antiphase patterns (|ϕ|>2π/3). The trend of lines show how the dominance of near-inphase and near-antiphase patterns change with increasing number of connections: inphase was increasingly preferred whereas antiphase diminished for connection number exceeding 4.

Section G. Relative phase distributions compared to chance level

To identify which patterns of phase coordination were significantly above chance level, confidence intervals (two-sided, for $\hat{p}$ <0.05, where 'hat' denotes Bonferroni correction for multiple comparison in 100 bins of an entire distribution) were computed from randomly permuted data (as detailed in Section D), and are shown as light-color bands in Fig E. A black bar marks wherever the real distribution is outside the confidence interval for three consecutive bins out of the hundred bins that make up the histogram. Within-group phase coordination shows significantly more near inphase patterns ($\phi \approx 0$) than chance levels ($\hat{p}$ <0.05, for 0 to 0.24π, Fig E(A1)), while between-group coordination, though with a hint

of inphase preference, was barely above chance ($\hat{p}$ <0.05, for 0.05π to 0.08π, Fig E(A2)). For low diversity (δf=0, 0.3 Hz, Fig E(B1-2)), the probability density near inphase was significantly above chance (highest peaks in blue and red curves). As diversity increased, the attractiveness of inphase patterns diminished, and beyond the critical diversity level (δf^*=0.5Hz) its statistical significance eventually vanished (δf=0.6 Hz, Fig E(B3)).

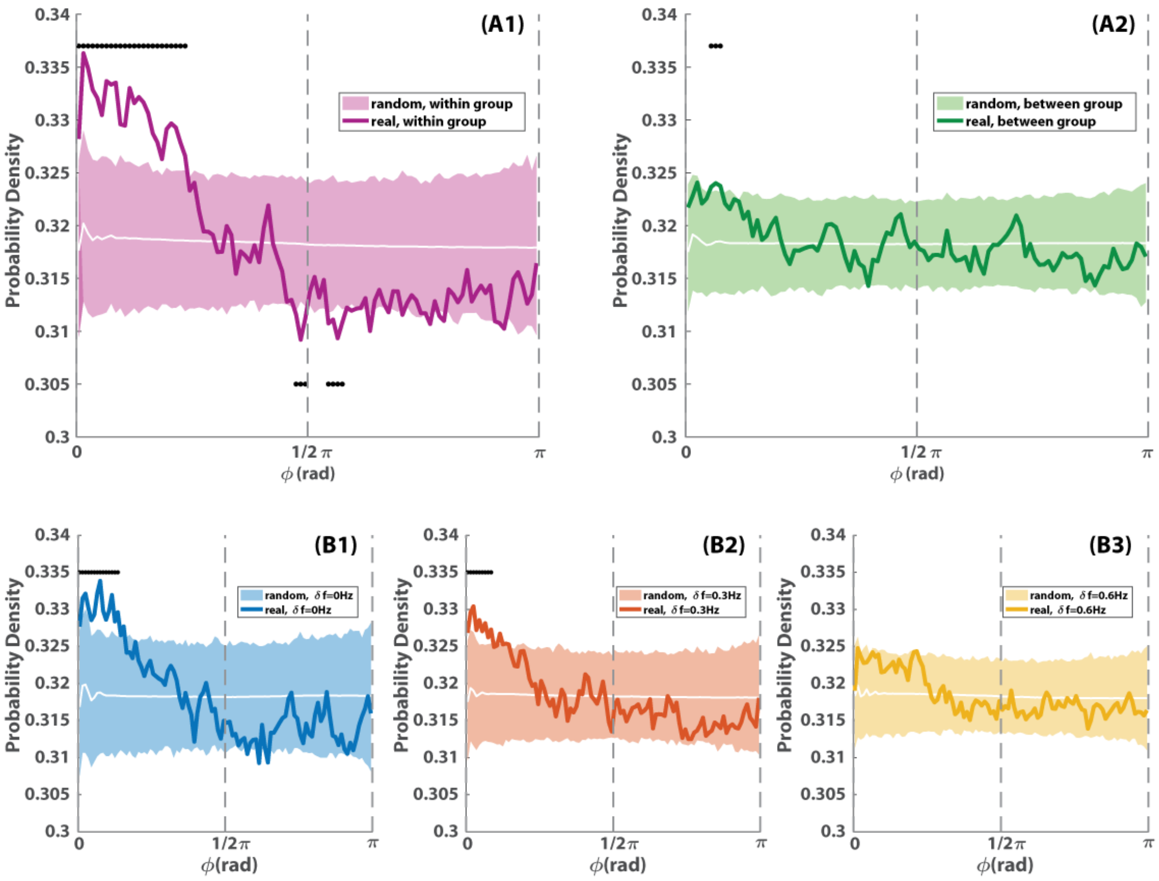

Fig E. Distributions of relative phase with respect to chance level. Within-group phase coordination (A1) shows more patterns near inphase are significantly above chance than between-group coordination (A2). At the aggregate level of ensembles, a tendency for inphase coordination is observed (B1-B3, solid lines), which is significantly different from chance (shaded areas) for lower diversity levels (B1, B2; black bar above indicates where more than 3 consecutive bins reached significance level $\hat{p}$ <0.05).

Section H. Phase relations are subject to internal and external changes in network structure

Using the onset and offset of each episode of strong coordination (as in Section F), we constructed a series of networks (i.e. graphs) representing the evolution of an ensemble's coordinative behavior during each trial of interaction (graphic examples see Fig 1A3, B4 and Fig 5D). Each node of a network represents a participant and an edge exists between two nodes if and only if the two participants are strongly coordinated at the time. Thus, we were able to detect changes in the coordinative structure or network (i.e. any two persons in the ensemble who were coordinated become uncoordinated or vice-versa) and to determine how dyadic relative phase was influenced by such changes. Fig F(A) shows distributions of the shift in dyadic relative phase between consecutive 2s windows: the blue curve depicts phase shifts in strongly coordinated dyads when there was no change in network (baseline; blue); the red curve depicts how much relative phase shifted in strongly coordinated dyads when other relations (edges) were forming or breaking up in the network (i.e. changes external to the dyad of interest; orange); and the yellow curve depicts how much phase shift occurred in dyads who were entering or leaving a strong coordination themselves (i.e. changes internal to the dyad of interest; yellow). Based on ANOVA, significant differences were found between the three conditions (Fig F(B); $F(2,663)=1165.33$, $p<0.001$). Relative phase shifted the most when the dyads themselves were changing from being coordinated (dwell) to uncoordinated (escape) or vice-versa (yellow; greater than blue and red, $p<0.001$, based on Tukey HSD). For strongly coordinated dyads (red), the phase relation was also shifted by changes elsewhere in the network to an extent significantly greater than baseline (i.e. no change in the network, blue; $p<0.001$, Tukey HSD). This result demonstrates quantitatively that dyadic coordination dynamics was influenced by the multiagent environment in which it is embedded.

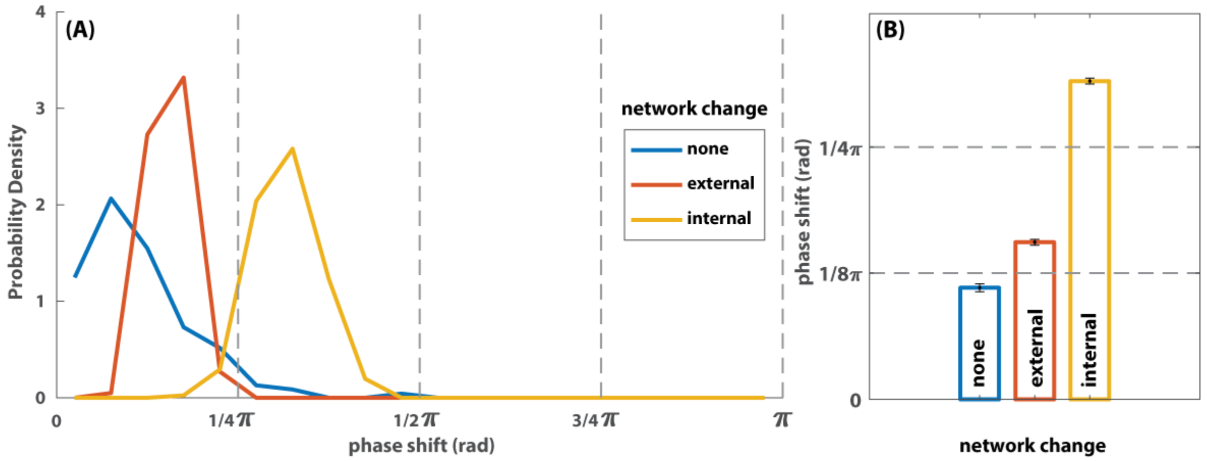

Fig F. Phase shift induced by internal and external changes of network structure. (A) Distribution of phase shift between consecutive 2s windows in dyads with strong relations: when (blue) there is no change in the network (all dyads un/coordinated remained un/coordinated), (red) the strongly coordinated dyad remained coordinated but changes occurred elsewhere in the network, and (yellow) the strongly coordinated dyad changed from being coordinated to uncoordinated or vice-versa. (B) compares the mean of distributions shown in (A) where error bars indicate the standard error of the mean. When there is a change in the network structure, both the dyads that remained coordinated and those that became (un)coordinated underwent a shift in relative phase significantly greater than baseline (i.e. when there was no change in the network structure, blue; all $p<0.001$), but the former experienced a smaller shift than the latter (red, yellow respectively; $p<0.001$).

Section I. Metastability in the multiagent environment

As exemplified in Fig 1 of the main text, participants engaged in metastable phase coordination. Metastably coordinated agents intermittently dwell at and escape from certain preferred phase patterns (i.e. coexistence of integration and segregation at dyadic level), which can lead to constant spatial reorganization (e.g. Fig 1, A3 and B4, Fig 5) as a flexible form of within-group integration. Here we want to emphasize that such spatial organization and reorganization is inseparable from the group's frequency organization. The time scale of a metastable relation between two agents depends on the difference between their natural frequencies (or frequency predispositions) – the greater the difference, the shorter and more recurrent the dwells (Tognoli and Kelso 2014). For example, in the trial illustrated in Fig 1B,

we showed that the shortest and most recurrent dwells in a group of four occurred between agents 4 and 3 (B1, blue). In Fig G, we show the corresponding frequency trajectories of these four agents. Notice that agent 3 (Fig. G, orange) tapped at a much higher frequency, on average, than the rest of the group; in contrast, agents 1, 2, 4 were much closer with each other in frequency, and their phase dwells lived on a longer time scale (Fig 1, B3, red and green). Furthermore, metastable phase coordination is accompanied by corresponding oscillations in instantaneous frequency: for a pair of agents to escape from a phase pattern, at least one of them must accelerate/decelerate to leave the common frequency, and for a pair to dwell in a particular phase pattern, two agents each at a distinct frequency must temporally accelerate/decelerate to converge to the same frequency (this property is used for a statistical analysis of metastability in Section J). This can be clearly seen in Fig G: agent 3 (orange) exhibited the most pronounced and rapid frequency oscillation, less so for agent 4 (yellow), and even less for agents 1 and 2 (red and pink). This is in stark contrast with the idealized picture of conventional synchronization where agents converge to and stick with the same frequency to maintain constant phase relations. Thus, metastability not only afford an overall integrated group flexible spatial organization but also preserve its frequency diversity – two sides of the same coin.

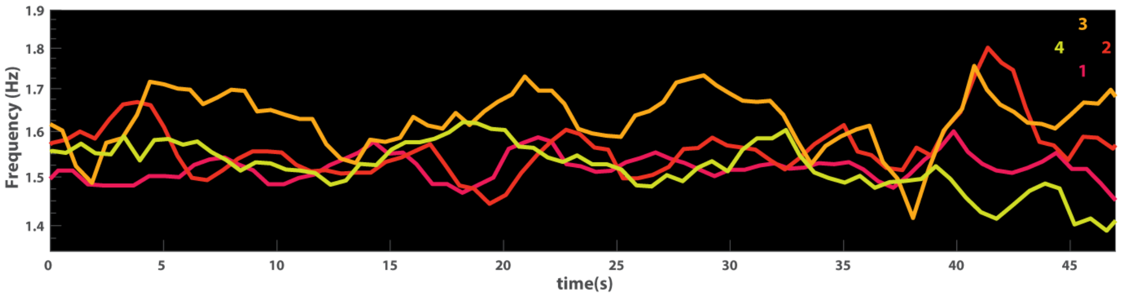

Fig G. Frequency variation in a group of metastably coordinated agents. This Fig shows instantaneous frequencies of agents whose phase coordination was illustrated in Fig 1B. The time series were smoothed with a 4-point moving average to capture the dynamics that best reveals spatial reorganization. Agent 3 had the highest tapping frequency, which oscillated up and down to join other agents at different times (e.g. around 17s, 25s and 33s), corresponding to the dwells illustrated in Fig 1B for relative phase between 3 and 4. Agent 4 oscillated between coordinating with 3 or 1-2, reflected in Fig 1B as short dwells in trajectories 1-4

and 4-3. Agents 1 and 2 were at the same frequency most of the time, reflected as the long dwell of 1-2 in Fig 1B.

The above case (Fig G) shows how a small group maintains long-term integration through constant transformation, but this may not be as easy for a larger group. Fig H(A,B) shows an example of eight-agent interaction where all agents were predisposed to the same frequency: A shows agents' frequency trajectories, and B shows the dynamics of their spatial organization as grouping dynamics (each column depicts which agents were phase coordinated as a group at a particular time, where agents in the same group were labeled with the same color; here "group" is equivalent to connected component in network structure). Early on in the trial, the ensemble of eight dwelled at very similar frequencies, although with constantly changing spatial configuration. This, however, did not last long as around 10-15s the big group began to diverge in frequency (Fig H(A)) and broke up into smaller, but more sustainable groups (e.g. pink and blue groups in Fig H(B)). This example demonstrates that when diversity is low, each agent has many potential partners to coordinate with: such potential relations may compete with each other, resulting in rapid switching between different partners and a lack of persistent spatiotemporal organization. To achieve more persistent grouping patterns, agents may diverge in their frequencies hence providing some separation protecting local structures. This example complements the high diversity case reported in the main text (Fig 5) in which spatial organization was much more persistent but not without sudden transitions – ensembles with high initial diversity had sufficient divergence to begin with. The grouping dynamics corresponding to Fig 5 is shown in Fig H(C) for visual comparison, in which the spatial organization is much more ordered and better sustained.

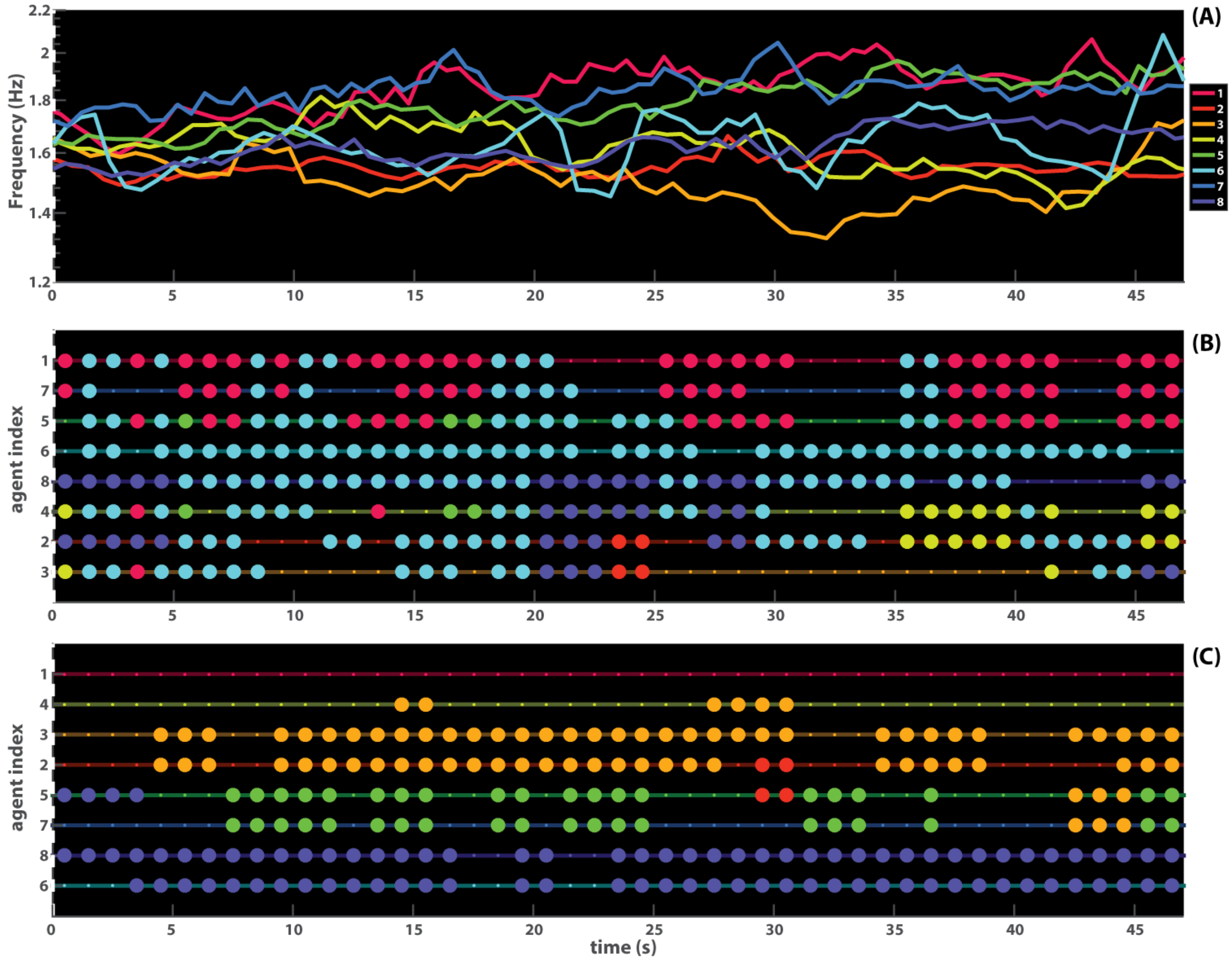

Fig H. Evolution of coordination dynamics among eight agents with low diversity (A-B) vs. high diversity (C). (A) Instantaneous frequencies of eight agents who were all paced with the same metronome. The frequency dispersion among agents gradually increased over the course of interaction. By the end of the trials, agents diverged into smaller groups. (B) Grouping dynamics among the eight agents with low initial diversity corresponding to (A). Each color represents a particular group, in which all members of the group were directly or indirectly connected through strong phase coordination as identified in Section F. Each row represents one agent whose group affiliation was denoted by the color of the circle (no circle means the agent was by him/her self), arranged, from top to bottom, from highest to lowest average frequency. The grouping (spatial configuration) changes rapidly early on in the trial but gradually forms a more stable structure later in the trial (agents are attracted to a three-group organization: 1-7-5, 6-8, and 4-2; see text). (C) For comparison, grouping dynamics among the eight agents with high initial diversity, corresponding to the example in Fig 5. The grouping (three groups/dyads: 3-2, 5-7, and 8-6) remained prevalent throughout the

interaction, but not without occasional reorganization, e.g. around 30s (see main text for details). That is, initial separation in frequency seem to favor the sustenance of lasting coordinative structures as in C, whereas initial similarity might create incidental opportunities for interaction that constantly perturb ongoing coordinative structures (A-B).

Section J. Quantification of metastability for statistical analysis

As discussed in Section I, metastable coordination entails fluctuations in agents' instantaneous frequency (e.g. Fig G, agent 3). If a phase relation between two agents is metastable, then as the relative phase (ϕ) goes through 2π (wrapping back to the same phase relation), there should be correspondingly oscillations in the relative frequency between the two agents (or equivalently oscillations in the derivative of ϕ). The degree of metastability was assessed based on this relation between the number of times ϕ wrapped through 2π (called the winding number), and the number of oscillations in relative frequency. We define a metastable index (MI) as

$$MI = P[\Delta F_{ij}]\left(\frac{wind(\phi_{ij})}{T}\right) \qquad \text{Eq.2}$$

where $\Delta F_{ij} = F_i - F_j$ is the relative frequency between participant *i* and *j* (normalized to Euclidean norm 1, mean removed, with an added small value ϵ = 10^{-4} Hz, explained later), $P[\Delta F_{ij}]$ is the power spectrum of ΔF_{ij} (in Decibels, dB), $wind(\phi_{ij}) := \frac{|\phi(T)-\phi(0)|}{2\pi}$ is the winding number, ϕ is the unwrapped relative phase and T is the length of the trial. This metric thus reflects the interplay between frequency fluctuation and phase wrapping in metastable relations. MI is high when metastable coordination is strong with a pronounced alternation of dwells and escape times (for each cycle of phase wrapping there is a corresponding oscillation of frequency difference), but low when the coordination is very weak (faint dwell/escape approaching uncoupled oscillators) or completely stable (thus not metastable). Completely stable phase-coordination corresponds to $P[\Delta F_{ij}](0)$ (i.e. no phase wrapping) which also corresponds to

the mean of ΔF_{ij}, thus we set mean of ΔF_{ij} to be ϵ so that in the power spectrum we have $P\left[\Delta F_{ij}\right](0) \approx -40dB$ (here ϵ must be non-zero, otherwise $P\left[\Delta F_{ij}\right](0) \rightarrow -\infty$).

To test whether MI truly reflects metastable coordination, we first validate MI with simulated data of dyadic coordination based on a well-tested theoretical model (extended HKB; see Section K for details). Based on existing empirical and theoretical studies of metastable coordination dynamics (Kelso, 1995; Tognoli and Kelso, 2014), we expect metastability to be high for weak/moderate coupling between agents with sufficiently large diversity in their frequency predispositions, and to be minimal when two agents do not couple to each other or are completely phase-locked to each other. Complete phase-locking results from a combination of strong coupling and low diversity. Were MI a valid metric, we should see a decrease of metastability with increasing coupling strength. First, 600 trials were simulated for dyads with zero coupling (a=0, b=0, thus no coordination), three levels of intrinsic frequency difference (i.e. diversity, δf=0, 0.3, 0.6 Hz, 200 trials for each level) and random initial conditions. Without coupling, the resulting MIs were -40.97±2.10 dB for all three levels of diversity – the minimal level of metastability as expected. Further, 1800 trials (200×3×3) were simulated for dyads with three levels of coupling (b=0.1, 0.4, or 0.8, with fixed a=1), three levels of diversity (mean δf=0, 0.3, 0.6 Hz, with 0.1 Hz variance), and random initial conditions. The parameters (black circles in Fig I) were so chosen to span the boundary between stable and metastable regimes of the model used to simulate coordination (yellow vs. blue areas in Fig I; see Section K for details).

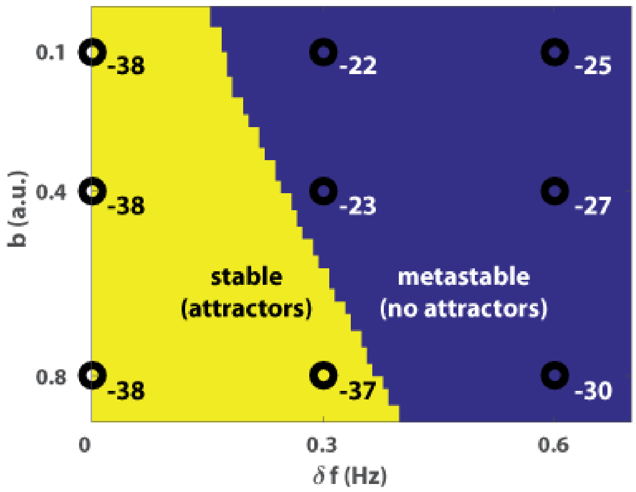

Fig I. Parameter space of the theoretical model used for simulation (extended Haken-Kelso-Bunz equations, Kelso et al, 1990, also see Section K). Vertical axis represents the coupling strength b and horizontal axis the frequency difference between two oscillatory components. Yellow area is the stable regime where there is at least one attractor of the system; blue area is the metastable regime where there is no attractor but attracting tendencies near certain phase patterns remain, giving rise to intermittent coordination (dwells and escapes). Black circles are parameter values chosen for the validation of MI. The number next to the circle indicates the average MI of simulated data based on that set of parameter values. MI successfully reflects the stable vs. metastable nature of the simulated coordination: near -40 dB in stable regime (yellow area) and between -30 to -20 dB (far from -40 dB) in metastable regime (blue).

From the simulated data, we found that, overall, MI decreased monotonically with increasing coupling strength (for b=0.1, 0.4, and 0.8, the MIs are -28.43±0.23 dB, -29.92±0.23 dB, and -35.07±0.23 dB respectively, not shown in Fig; MANOVA main effect, $F(2,1971)=229.79$, $p<0.0001$; post hoc test with Tukey HSD, all $p<0.0001$); and MI is the lowest for δf=0Hz (-38.19±0.23 dB, not shown, main effect, $F(2,1791)=707.63$, $p<0.0001$; post hoc test, greater than for δf=0.3, 0.6Hz, both $p<0.0001$), while MI for δf=0.3 and 0.6Hz were not significantly different from each other (-27.78±0.23 dB and -27.44±0.23 dB respectively, not shown, post hoc test, $p>0.05$). A breakdown of all conditions is shown in Fig J(B) (MANOVA, interaction effect, $F(4,1791)=108.43$, $p<0.001$). For weak coupling (b=0.1, 0.4), metastability was greatest when diversity was moderate (δf=0.3Hz, orange); slightly lowered when

diversity became too high (δf=0.6Hz, yellow), but dropped close to minimum for low diversity (δf=0Hz, blue). When the coupling is strong (b=0.8), dyads with moderate diversity also lost metastability, indicating complete phase-locking. As expected from existing studies of metastability weak coupling and moderate diversity led to greater MI while strong coupling diminished MI, thereby showing MI is a valid metric of metastability.

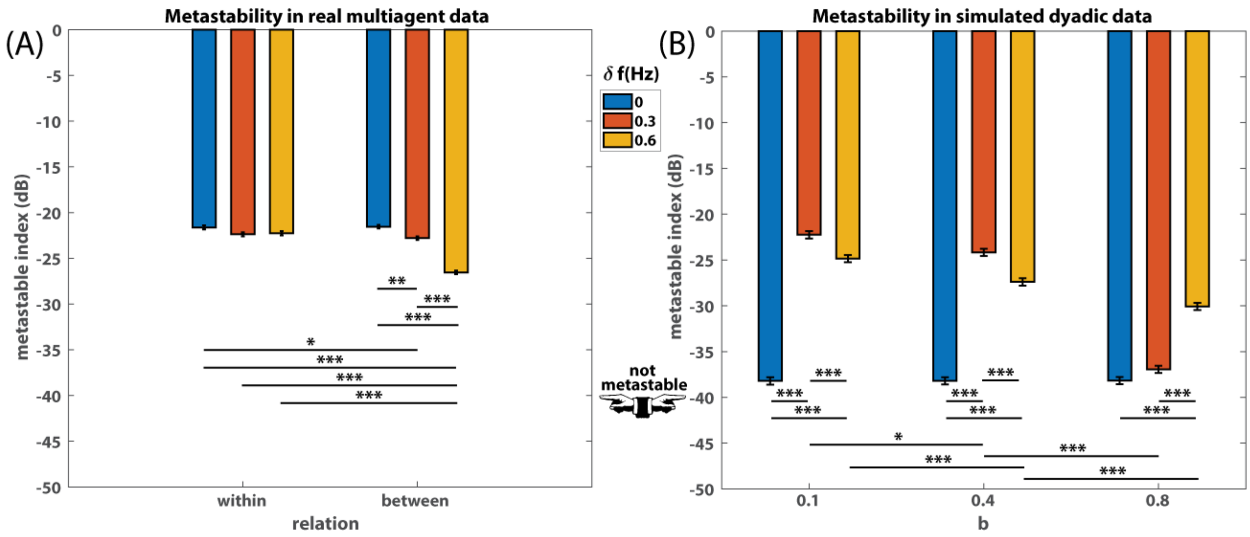

Fig J. Metastable coordination within and between groups. (A) Metastability in real data collected in the present experiment. (B) Metastability in simulated data of dyadic coordination. (* p<0.05; ** p<0.01; ***p<0.001)

Applying MI to the actual data of the present experiment, we found main effects (not shown in Fig) for both grouping relation (whether metastable coordination occurs within- or between-group, $F(1,7246)=77.67$, $p<0.0001$) and diversity δf ($F(2,7246)=89.21$, $p<0.0001$): within-group coordination (MI=-22.09±0.13 dB) exhibits greater metastability than between group (-23.63±0.11 dB; post hoc test, Tukey HSD, $p<0.0001$), and the metastable index monotonically decreases with increasing diversity (for δf=0Hz, -21.58±0.15 dB, for δf=0.3Hz, -22.58±0.15 dB, and for δf=0.6Hz, -24.40±0.15 dB; post hoc test, all $p<0.0001$). MI was far from the minimal value (≈ -40dB) in every condition. This indicates that the phase coordination present in this experiment is overwhelmingly metastable. A significant interaction

effect between grouping relation and diversity was also found ($F(2,7246)=62.39$, $p<0.0001$) as shown in Fig J(A). There was no significant change in the level of metastability for within-group coordination with increasing intergroup difference (host hoc test, $p>0.05$; Fig J(A), left), nor was any within-group metastability significantly different from between-group metastability when diversity was minimal (δf=0 Hz, Fig J(A), right, blue bar; $p>0.05$). With increasing diversity (δf=0.3, 0.6Hz, Fig J(A), right, orange and yellow bars), metastability in between-group coordination decreases (though still far from minimum -40 dB). Overall, these results suggest that in this experiment metastable relations, rather than stable ones, are the building blocks of spontaneous multiagent coordination, even in the case of lowest diversity (δf=0 Hz).

Section K. Simulations of dyadic interaction

Dyadic rhythmic coordination has been extensively studied in existing research (see main text). The extended Haken-Kelso-Bunz equation (Fuchs et al 1996; Kelso, et al., 1990) is a well-tested model that captures the essential dynamics of dyadic coordination:

$$\dot{\phi} = \Delta\omega - a\sin\phi - 2b\sin 2\phi \qquad \text{Eq. 1}$$

where ϕ is the relative phase between two oscillators, $\dot{\phi}$ is the rate of change of relative phase, $\Delta\omega$ is the difference between the two oscillators' natural frequency, and the ratio b/a reflects the coupling strength. Numeric solutions (2400 simulated 50s trials) were obtained using MATLAB (ode45), which were used to validate Metastable Index (MI, for details see Section J) and to produce results shown in Fig J(B).

Section L. Main effects of experimental conditions on phase-locking

Overall, within-group dyads exhibited greater phase-locking than between-group dyads (main effect, $F(1,7246)=604.7$, $p<0.001$); diversity (δf) weakened phase-locking (main effect, $F(1,7246)=338.6$; post hoc test, all $p<0.001$).

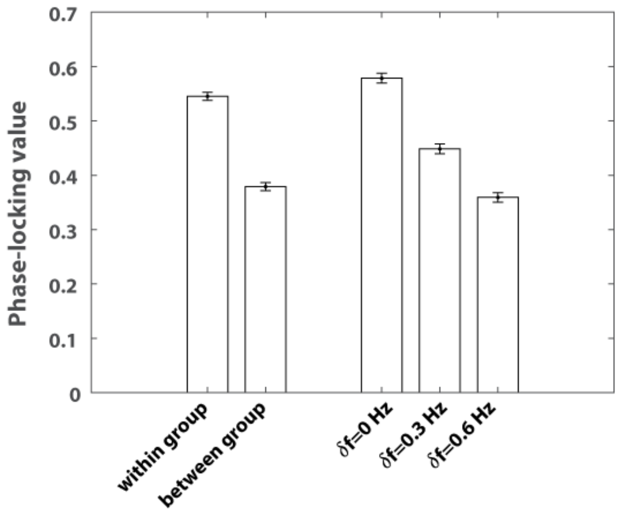

Fig K. Main effects of grouping relation (left cluster) and intergroup difference (right cluster) on phase coordination in MANOVA.

SI References

Fuchs, A., Jirsa, V. K., Haken, H., & Kelso, J. A. S. (1996). Extending the HKB model of coordinated movement to oscillators with different eigenfrequencies. *Biological Cybernetics*, *74*(1), 21–30. https://doi.org/10.1007/BF00199134

Kelso, J. A. S., Del Colle, J. D., & Schöner, G. (1990). Action-perception as a pattern formation process. In M. Jeannerod (Ed), *Attention and performance 13: Motor representation and control* (Vol. 45, pp. 139–169). Hillsdale, NJ, US: Lawrence Erlbaum Associates, Inc.

Tognoli, E., & Kelso, J. A. S. (2014). The metastable brain. *Neuron*, *81*(1), 35–48. https://doi.org/10.1016/j.neuron.2013.12.022
